# Type-3 Hyaluronan Synthase Attenuates Tumor Cells Invasion in Human Mammary Parenchymal Tissues

**DOI:** 10.3390/molecules26216548

**Published:** 2021-10-29

**Authors:** Wen-Jui Lee, Shih-Hsin Tu, Tzu-Chun Cheng, Juo-Han Lin, Ming-Thau Sheu, Ching-Chuan Kuo, Chun A. Changou, Chih-Hsiung Wu, Hui-Wen Chang, Hang-Lung Chang, Li-Ching Chen, Yuan-Soon Ho

**Affiliations:** 1 Ph.D. Program for Neural Regenerative Medicine, College of Medical Science and Technology, Taipei Medical University, Taipei 110, Taiwan; sam19871119@hotmail.com; 2 Ph.D. Program for Neural Regenerative Medicine, College of Medical Science and Technology, National Health Research Institutes, Miaoli County 350, Taiwan; 3 Breast Medical Center, Taipei Medical University Hospital, Taipei 110, Taiwan; drtu@h.tmu.edu.tw; 4 Taipei Cancer Center, Taipei Medical University, Taipei 110, Taiwan; 5 Department of Surgery, School of Medicine, College of Medicine, Taipei Medical University, Taipei 110, Taiwan; 6 Graduate Institute of Medical Sciences, College of Medicine, Taipei Medical University, Taipei 110, Taiwan; d119096007@tmu.edu.tw; 7 Program for Cancer Molecular Biology and Drug Discovery, College of Medical Science and Technology, Taipei Medical University and Academia Sinica, Taipei 110, Taiwan; d621100001@tmu.edu.tw; 8 Department of Pharmaceutical Sciences, Taipei Medical University, Taipei 110, Taiwan; mingsheu@tmu.edu.tw; 9 Institute of Biotechnology and Pharmaceutical Research, National Health Research Institutes, Zhunan 350, Taiwan; cckuo@nhri.org.tw; 10 The PhD Program for Translational Medicine, College of Medical Science and Technology, Taipei Medical University, Taipei 110, Taiwan; austinc99@tmu.edu.tw; 11 The PhD Program for Cancer Molecular Biology and Drug Discovery, College of Medical Science and Technology, Taipei Medical University and Academia Sinica, Taipei 110, Taiwan; 12 The Core Facility Center, Office of Research and Development, Taipei Medical University, Taipei 110, Taiwan; 13 Department of General Surgery, En Chu Kong Hospital, New Taipei City 110, Taiwan; chwu@tmu.edu.tw; 14 Department of Laboratory Medicine, Taipei Medical University Hospital, Taipei 110, Taiwan; g160090005@tmu.edu.tw; 15 Department of General Surgery, En Chu Kong Hospital, New Taipei City 237, Taiwan; changhl0321@gmail.com; 16 TMU Research Center of Cancer Translational Medicine, Taipei Medical University, Taipei 110, Taiwan; 17 Graduate Institute of Neural Regenerative Medicine, College of Medical Science and Technology, Taipei Medical University, Taipei 110, Taiwan; 18 School of Medical Laboratory Science and Biotechnology, College of Medical Science and Technology, Taipei Medical University, Taipei 110, Taiwan

**Keywords:** breast cancer, tumor microenvironment, hyaluronan synthase 3, tubulin acetylation, autophagy

## Abstract

The microenvironment for tumor growth and developing metastasis should be essential. This study demonstrated that the hyaluronic acid synthase 3 (HAS3) protein and its enzymatic product hyaluronic acid (HA) encompassed in the subcutaneous extracellular matrix can attenuate the invasion of human breast tumor cells. Decreased HA levels in subcutaneous Has3-KO mouse tissues promoted orthotopic breast cancer (E0771) cell-derived allograft tumor growth. MDA-MB-231 cells premixed with higher concentration HA attenuate tumor growth in xenografted nude mice. Human patient-derived xenotransplantation (PDX) experiments found that HA selected the highly migratory breast cancer cells with CD44 expression accumulated in the tumor/stroma junction. In conclusion, HAS3 and HA were detected in the stroma breast tissues at a high level attenuates effects for induced breast cancer cell death, and inhibit the cancer cells invasion at the initial stage. However, the highly migratory cancer cells were resistant to the HA-mediated effects with unknown mechanisms.

## 1. Introduction

Breast cancer is the most common cancer in women, and the second leading cause of death worldwide [1]. More than 2 million new breast cancer cases are diagnosed each year, and approximately 626,679 deaths occur annually [2]. Hyaluronic acid (hyaluronan, HA) is an anionic, nonsulfated glycosaminoglycan synthesized and distributed within connective, epithelial, and neural tissues [3]. HA is synthesized by a class of integral membrane proteins called hyaluronan synthases (HAS), of which vertebrates have three: HAS1, HAS2, and HAS3. The HAS enzymes synthesize HA by adding glucuronic acid and N-acetylglucosamine to a polysaccharide backbone. After this process is complete, HA is then extruded through the cell membrane into the extracellular space via the ATP-binding cassette (ABC) transporter [4].

A previous study demonstrated that EGFP-HAS3-overexpressing melanoma (MV3) cells synthesized HA with a molecular weight (MW) of primarily over 250 kDa. The synthesis of high-MW HA by HAS3 overexpression significantly reduced MV3 cell migration and proliferation and caused cell growth cycle arrest at the G0/G1 phase [5]. Interestingly, another study found that tumors with distinct margins showed more prominent HAS3 expression in stromal cells [6]. HAS3 is responsible for synthesizing unbranched glycosaminoglycan HA, an essential constituent of the ECM in breast tissue [7]. The antitumor activity of high-MW (117 kDa) HA surrounding the ECM space in breast tissues was tested by an in vitro study, which demonstrated that HA inhibited the proliferation of 4T1 and BT549 breast cancer cells through increasing p53- and p21-mediated apoptotic signals [8]. The anti-invasive effects of HAs with different MWs were assessed in breast cancer (SKBR3) cells. The results suggested that low MW (35 kDa) HA significantly promoted cell invasion, and that high MW (117 kDa) HA inhibited cell invasion at all concentrations tested [9]. The HAS3 in a human neuroblastoma cancer cell line (N2a) was induced by serum starvation (<2.5% FBS) in our previous study. The HAS3 protein significantly induced N2a cancer cell differentiation and caused autophagic cell death [10].

In this study, we first demonstrated that the mRNA and protein level of HAS3 were higher in normal breast epithelial cells (MCF-10A, MCF-12A, and 184A1) and adjacent normal stroma in corresponding tumor tissues. This study explains the possible antitumor mechanism of clinically observed high HAS3 protein levels in normal breast tissue ECM. We further found that malignant breast cancer cells established from the mouse E0771-allografted tumor lung metastasis site (E0771-M) were resistant to HA. Our study first explores the antitumor effects of HAS3 in tumor-adjacent ECM. This study’s results provide the molecular mechanisms of the HA potential for applying breast tumor therapy.

## 2. Material and Methods

### 2.1. Cell Culture

HCC1419 (ATCC^®^ CRL-2326™), MDA-MB-453 (ATCC^®^ HTB-131™), MDA-MB-468 (ATCC^®^ HTB-132™), HCC38 (ATCC^®^ SC-CRL-2314™), HCC1395 (ATCC^®^ SC-CRL-2324™), BT-549 (ATCC^®^ HTB-122™), BT-474 (ATCC^®^ HTB-20™) SK-BR-3 (ATCC^®^ HTB-30™), AU565 (ATCC^®^ CRL-2351™), UACC-893 (ATCC^®^ CRL-1902™), Hs 587T (ATCC^®^ HTB-126™), MDA-MB-231 (ATCC^®^ HTB-26™), BT-20 (ATCC^®^ HTB-19™), HCC1937 (ATCC^®^ CRL-2336™), MDA-MB-436 (ATCC^®^ HTB-130™), MCF-7 (ATCC^®^ HTB-22™), T-47D (ATCC^®^ HTB-133™), ZR-75-1 (ATCC^®^ CRL-1500™), MCF 10A (ATCC^®^ CRL-10317™), MCF-12A (ATCC^®^ CRL-10782), and 184A1 (ATCC^®^ CRL-8798) cells were purchased from the American Type Culture Collection (ATCC, Northern, VA, USA). The E0771 (940001) murine breast cancer cell line was purchased from CH3 BioSystems Company (Amherst, NY, USA). The cells were cultured in Dulbecco’s Modified Eagle Medium/Nutrient Mixture F-12 (DMEM/F12, Gibco, CA, USA) supplemented with 10% heat-inactivated fetal bovine serum (FBS; Gibco, Amarillo, TX, USA) and 50 U/mL penicillin/streptomycin/neomycin (Invitrogen, Waltham, MA, USA) in a humidified (5% CO_2_, 37 °C) incubator. All human breast tumor samples (*n* = 332) were obtained as specimens from anonymous donors from Taipei Medical University Hospital, Taipei, Taiwan, as approved by the Institutional Review Board (IRB) and ethics committee of the institution (P102025). On histological inspection, all patient samples consisted of more than 80% tumor tissue. All samples (each paired tumor tissue vs. normal tissue) were collected and categorized according to clinical characteristics, such as age.

### 2.2. Primary Culture of HB-TDFs

Human breast cancer specimens were collected from the TMU Biobank (TMU-IRB, P102025, Taipei, Taiwan). Fresh breast cancer tissue was dissected using the aseptic technique, following which, the tissue was minced, trypsinized (addition of trypsin, a proteolytic enzyme that facilitates the breakdown of tissue into single cells), and seeded into T25 tissue culture flasks. The cells were grown at 37 °C under 5% CO_2_ in complete RPMI 1640 medium (Gibco, Amarillo, TX, USA), supplemented with 10% heat-inactivated FBS (Gibco, Amarillo, TX, USA) and 50 U/mL penicillin/streptomycin/neomycin (Invitrogen, Waltham, MA, USA) in a humidified (5% CO_2_, 37 °C) incubator. Fibroblast characteristics were confirmed via morphology and flow cytometry analysis with antibodies against Vimentin (ab20346, Abcam, Cambridge, UK) and alpha-smooth muscle actin (α-SMA, ab7817, Abcam, Cambridge, UK) [11].

### 2.3. Cell Proliferation Assay

Cell proliferation was determined using the 3-(4,5-dimethylthiazol-2-yl)-2,5-diphenyltetrazolium (MTT) assay. Sh-HAS3- and Sc-HAS3-transfected MDA-MB-231 cells were seeded at a density of 8000 cells per well in 96-well plates and cultured at 37 °C overnight for 24, 48, and 72 h [12]. This assay was repeated four times with duplicate samples.

### 2.4. HAS3 Gene RNA Interference Experiments

HAS3 expression was ablated in MDA-MB-231 cells with small interfering RNA (siRNA). A scrambled siRNA sequence (scRNA) was used as a control. After BLAST analysis to verify the absence of significant sequence homology with other human genes, the selected sequences were inserted into BglII- and HindIII-cut pSUPER vectors to generate the pSUPER-Sh-HAS3 and pSUPER-Sc-HAS3 vectors. The identities of all constructs were confirmed by DNA sequence analysis. The primer sequences were designed as follows: pSUPER-Sh-HAS3 sense sequence 5′-GATCCCCGTGGTCATGGTGGTGGATGTTCAAGAGACATCCACC ACCATGACCACGGG-3′, antisense sequence 5′-AGCTTAAAAAGTGGTCATGG TGGTGGATGTCTCTTGAACATCCACCACCATGACCACGGG-3′ and pSUPER-Sc-HAS3 sense sequences 5′-GATCCCCGATATACATGCTCGCGCGCTTCAAGAGAG CGCGCGAGCATGTATATCTTTTA-3′, antisense sequence 5′-AGCTTAAAAAGAT ATACATGCTCGCGCGCTCTCTTGAAACCGTCAGCAGGAAGAGGAGGG-3′.

### 2.5. Immunohistochemistry (IHC) Analysis

HAS3 protein expression in breast tumor tissues was detected by immunohistochemistry (IHC). Paraffin-embedded breast tumor tissues were cut into 8-μm sections. The sections were preincubated in 3% H_2_O_2_ and 0.3% Triton X-100 before microwaving for antigen retrieval. For HAS3 immunostaining, sections were microwaved in Tris buffer (pH 6) for 10 min. Following this step, the antigenicity of the tumor cells in sections was blocked in 5% horse serum (Chemicon, Temecula, CA, USA) for 30 min, and subsequently incubated with diluted (1:400) HAS3 (A6617, Abclonal, Woburn, MA, USA)-specific antibody for 2 h at room temperature. Following incubation with the primary antibody, staining was developed with the streptavidin-biotin-peroxidase method using an LSAB 2 kit purchased from Dako (Carpinteria, CA, USA). Briefly, sections were washed in phosphate-buffered saline (PBS) and incubated with biotinylated anti-rabbit secondary antibody. The samples were washed again in the same buffer and incubated in streptavidin-biotin-peroxidase complex. Staining was completed after incubation with a substrate-chromogen solution. The duration of incubation in solution with 3,3′-diaminobenzidine was determined by low-power microscopic inspection. Slides were then washed, dehydrated, and mounted on coverslips using a mixture of distyrene, plasticizer, and xylene (DPX mounting medium) (44581, Sigma-Aldrich, St. Louis, MO, USA). Both adjacent sections on the same slides were counterstained with hematoxylin for general histological orientation.

### 2.6. Measurement of Serum HA Concentrations

Serum HA levels were measured with an HA-binding protein-based enzyme-linked immunosorbent assay (ELISA)-like quantitative kit (HA Test Kit; Corgenix, Broomfield, CO, USA) according to the manufacturer’s instructions in duplicate; a coefficient of variation (CV) <10% was considered acceptable. The absorbance was measured on a fluorescence spectrophotometer (F4500, Hitachi, Tokyo, Japan), and plotted by polynomial regression against the corresponding concentration on the standard curve [13].

### 2.7. Determination of the Molecular Mass of HA by Sandwich ELISA-like Assay in Conditioned Medium Harvested from MEF and MEF-Has3-KO Cells

Conditioned medium (15 mL) was harvested from MEF and MEF-Has3-KO cells cultured by standard protocols for 24 h. After that, samples with different MW cutoffs were concentrated according to the standard protocol by using the following ultracentrifugal filters: Amicon^®^ Ultra-15-100 kDa (UFC910008, Merck Millipore, Burlington, MA, USA), Amicon^®^ Ultra-15-50 kDa (UFC905008, Merck Millipore, Burlington, MA, USA), Amicon^®^ Ultra-15-30 kDa (UFC903008, Merck Millipore, Burlington, MA, USA), and Amicon^®^ Ultra-15-3 kDa (UFC900308, Merck Millipore, Burlington, MA, USA). The concentration of HA (ng/mL) in concentrated samples was determined by using an HA ELISA-like assay [14].

### 2.8. In Vitro Cell Migration (Transwell) Assay

An in vitro cell migration assay was performed using 24-well Transwell inserts (8 μm) (Corning, Midland, NY, USA). Briefly, Transwell inserts were seeded with 2 × 10^4^ MDA-MB-231 cells, and the lower chambers were filled with HA (40583, 73641, 41897, 96144, 63357, 08185, 75574, 42686, Sigma-Aldrich, Burlington, MO, USA) at different concentrations. The assay was carried out at 37 °C for 4 h. After fixation, the cells were stained with 0.5% crystal violet (Sigma-Aldrich, Burlington, MO, USA) and 10 μg/mL Hoechst (Sigma-Aldrich, Burlington, MO, USA). The proportion of migrated cells in the Transwell inserts in all fields were calculated by microscopy (DMI 4000B, Leica, Wetzlar, Germany), and the calculation was repeated in three independent experiments.

### 2.9. RNA Extraction and Quantitative Reverse Transcription PCR

According to the manufacturer’s protocol, total RNA was isolated from human cell lines and breast tumor tissue samples from patients using TRIzol (Invitrogen, Waltham, MA, USA). A LightCycler thermocycler (LC 2.0, Roche Molecular Biochemicals, Mannheim, Germany) was used for real-time quantitative PCR. The fluorescence intensity of the HAS3 mRNA was measured and normalized to β-glucuronidase expression using built-in software (Roche LightCycler version 4, Indianapolis, IN, USA). The calculation of copy number followed a previous study [15]. The copy concentration was calculated using the following equation:

$$\mathrm{DNA}\;\left(\mathrm{copy}\right)=\frac{6.02\;\times \;10^{23}\;\left({\mathrm{copies}\;\mathrm{mol}}^{-1}\right)\;\times \;\mathrm{DNA}\;\mathrm{amount}\;(g)\;}{\mathrm{DNA}\;\mathrm{length}\;\left(\mathrm{bp}\right)\;\times \;660\;(g\;{\mathrm{mol}}^{-1}{\mathrm{bp}}^{-1})}$$

Relative calculation was performed according to the following equation:

$$\mathrm{Target}\;\mathrm{to}\;\mathrm{reference}\;\mathrm{ratio}=\frac{{\left(1+E_{t}\right)}^{-\Delta C_{T,t}}}{{\left(1+E_{r}\right)}^{-\Delta C_{T,t}}}$$

*E_t_*  =  amplification efficiency of the target gene, *E_r_*  =  amplification efficiency of the reference gene, Δ*C_T,t_*  =  target C_T_ in sample − target C_T_ in the calibrator, Δ*C_T,r_ * =  reference C_T_ in the sample − reference C_T_ in the calibrator.

The primer sequences were designed as follows: HAS1 primer sense sequence: 5′-GGAGGGTGCTGACCATC-3′ and HAS1 primer antisense sequence: 5′-TCCAGGTACGCGAAGAG-3′; HAS2 primer sense sequence: 5′-GCTGAACAAGATGCATTGTGAGA-3′ and HAS2 primer antisense sequence: 5′-ATAGGCAGCGATGCAAAGGG-3′; HAS3 primer sense sequence: 5′-CAGTTCATCCACACGGAAA-3′ and HAS3 primer antisense sequence: 5′-CGCAAGTAGTCAGGGTC-3′; Has1 primer sense sequence: 5′-GATGACAGGCACCTCAC-3′ and Has1 primer antisense sequence: 5′-CCACTCTCGGAAGTAAGATT-3′; Has2 primer sense sequence: 5′-CCCTATGGTTGGAGGTG-3′ and Has2 primer antisense sequence: 5′-CAGAGGACCGCTTATGC-3′; Has3 primer sense sequence: 5′-TCTCTGTGGTTCCATAAGC-3′ and Has3 primer antisense sequence: 5′-ATAGGTAGCCTTGATAATGCC-3′; Gapdh primer sense sequence: 5′-CCATCTTCCAGGAGCGA-3′ and Gapdh primer antisense sequence: 5′-GTTCACACCCATCACAAAC-3′ GUS primer sense sequence: 5′-AAACAGCCCGTTTACTTGAG-3′ and GUS primer antisense sequence: 5′-AGTGTTCCCTGCTAGAATAGATG-3′.

### 2.10. Protein Extraction and Western Blotting

For protein extraction, cells were washed twice with ice-cold PBS and lysed on ice in Golden lysis buffer (20 mM Tris-HCl (pH 8.0), 137 mM NaCl, 5.95 mM EDTA, 5 mM EGTA, 10 mM NaF, 1% Triton X-100, and 10% glycerol) supplemented with protease inhibitors (Roche, Indianapolis, IN, USA) and phosphatase inhibitors (Sigma-Aldrich, St. Louis, MO, USA). The proteins were separated via 12% sodium dodecyl sulfate-polyacrylamide gel electrophoresis (SDS-PAGE) and transferred to polyvinylidene fluoride membranes. Specific antibodies against HAS3 (SAB2101014, Sigma-Aldrich, St. Louis, MO, USA), p21 (GT8611, GeneTex, Irvine, CA, USA), α-tubulin (GT114, GeneTex, Irvine, CA, USA), ATG5 (GTX113309, GeneTex, City, CA, USA), LC3 (GTX127375, GeneTex, Irvine, CA, USA), and GAPDH (sc-47724, Santa Cruz Biotechnology, Dallas, TX, USA) were diluted 1:2000 in Tris-buffered saline/Tween 20, and the membranes were incubated for 2 h at room temperature. Horseradish peroxidase-conjugated anti-mouse IgG (sc-2354, Santa Cruz Biotechnology, Dallas, TX, USA) and anti-rabbit IgG (sc-2004, Santa Cruz Biotechnology, Dallas, TX, USA) secondary antibodies were diluted 1:4000 and incubated with the membranes for 1 h at room temperature.

### 2.11. Immunofluorescence Staining and Confocal Microscopy

Cells were fixed with 4% paraformaldehyde in PBS for 15 min at room temperature and permeabilized with 0.1% Triton X-100 in PBS for 5 min at room temperature. Samples were blocked for 30 min in PBS with 2% bovine serum albumin (BSA). Primary antibodies against β-tubulin (GTX101279, GeneTex, Irvine, CA, USA), γ-tubulin (GTX113286, GeneTex, Irvine, CA, USA), acetyl-α-tubulin (GTX16292, GeneTex, Irvine, CA, USA), and HAS3 (SAB2101014, Sigma-Aldrich, St. Louis, MO, USA) were diluted 1:100 in PBS with 1% BSA, and then incubated with the cells for 2 h at room temperature. AffiniPure goat anti-mouse FITC (15-095-003, Jackson ImmunoResearch, West Grove, PA, USA) and AffiniPure goat anti-rabbit rhodamine (111-025-144, Jackson ImmunoResearch, West Grove, PA, USA) secondary antibodies were diluted 1:50. Slides were incubated with secondary antibodies for 1 h at room temperature. Samples were mounted with VECTASHIELD Antifade Mounting Medium (H-1000, Vector Laboratories, Burlingame, CA, USA) and imaged via confocal microscopy (DMI 6000B CS, Leica, Wetzlar, Germany).

### 2.12. Transfection and Electroporation of Cells

Briefly, 1.5 × 10^5^ cells were washed twice with PBS and mixed with pSUPER-Sh-HAS3, pSUPER-Sc-HAS3, HAS3-EYFP-pcDNA3.1, or α-tubulin-AmCyan (10 μg) plasmid. Two pulses were applied for a duration of 20 milliseconds under a fixed voltage of 1.2 kV on an MP-100 pipette-type microporator (Digital Bio, Seoul, Korea). The transfection efficiency was determined by fluorescence microscopy (DMI 4000B, Wetzlar, Germany) and confocal microscopy (DMI 6000B CS, Leica, Wetzlar, Germany). The primer sequences were designed as follows: HAS3-EYFP-pcDNA3.1 sense sequence: 5′-ATTAAGCTTATGCCGGTGCAGCTGACGACA-3′, antisense sequence: 5′-TGAGAATTCCACACCTCAGCAAAAGCCA-3′ and α-tubulin-AmCyan sense sequence: 5′-ATAGCTAGCATGCGTGAGTGCATCTCCATC-3′ and antisense sequence: 5′-GAAACCGGTGTATTCCTCTCCTTCTTCCTC-3′.

### 2.13. Transmission Electron Microscopy (TEM)

After transfection with HAS3, MDA-MB-231 cells were seeded on chamber slides for 24 h. Samples were washed with 0.1 M cacodylate buffer in artificial seawater (ASW) and fixed in 0.1 M cacodylate buffer in ASW containing 2.5% glutaraldehyde for 1 h at room temperature. The cells were postfixed in 1% osmium tetroxide in ASW for 90 min. Subsequently, the samples were dehydrated through a graded ethanol series and embedded in epoxy resin overnight at 60 °C. Images were acquired via TEM (HT-7700, Hitachi, Tokyo, Japan).

### 2.14. Flow Cytometry Cell Cycle Analysis

MDA-MB-231 cells were cultured to 80% confluence and then synchronized in DMEM/F12 (Gibco, Amarillo, TX, USA), supplemented with 0.04% heat-inactivated FBS (Gibco, Amarillo, TX, USA). After synchronization, the medium was replaced with 10% FBS, and then the cells were harvested after 0, 3, 6, 9, 12, 15, 18, 21, 24, 27, 30, and 33 h. Cells were fixed with 70% cold ethanol for one hour and then treated with RNase A (Sigma-Aldrich, Saint Louis, MO, USA) in a 37 °C water bath for 30 min. After RNase A treatment, all the samples were stained with propidium iodide (PI) (Sigma-Aldrich, Saint Louis, MO, USA) at room temperature for 15 min. A total of 10,000 cells from each sample were detected by flow cytometry with a FACSCalibur (BD, Franklin Lakes, NJ, USA).

### 2.15. Fluorescence Lifetime Imaging (FLIM) Microscopy

FLIM was carried out using confocal microscopy (DMI 6000B CS, Leica, Wetzlar, Germany). The AmCyan fluorescence lifetime was measured upon 2-photon excitation at 405 nm. A 495 nm long pass filter was used to separate the blue and green fluorescence channels. AmCyan emission was detected with a blue filter (BP 420) in one channel, and enhanced yellow fluorescent protein (EYFP) emission was detected with a green filter (BP 500). Images were acquired using a 63× oil objective. Images with a resolution of 512 × 512 pixels were collected with a minimum of 1000 counts per pixel, which requires integrating at least 30 frames with a pixel dwell time of 25.6 us/pixel. The temperature was set to 37 °C throughout the experiment conducted in a 5% CO_2_ atmosphere.

### 2.16. Generation of Has3-KO Mice

Complex alleles lacking murine HAS3 were generated using improved genome editing via CRISPR-Cas9 technology. In general, murine HAS3 gene function was disrupted by frameshift deletions in exon 3. Two single guide RNAs (sgRNAs) were designed to disrupt the critical exon 3 (knockout). The sgRNAs were synthesized and then complexed with transactivating RNA (tracrRNA) and the Cas9 protein to form a ribonucleoprotein (RNP) complex. The RNPs were microinjected in situ with a long single-stranded oligonucleotide repair template into a single-cell embryo. The progenies were genotyped to confirm successful editing of the HAS3 gene.

### 2.17. Preparation of Mouse Embryonic Fibroblasts (MEFs)

To produce MEFs for the experiment, day 14 embryos were obtained from pregnant C57B6/J and C57B6/J-Has3-KO mice. After removing the head and red organs, the torso was minced and digested with 0.1% trypsin and 0.1% collagenase for 30 min at 37 °C. The cells were seeded onto 10-cm cell culture dishes and split once at 1:4 before being frozen.

### 2.18. In Vivo Human Breast Cancer Xenograft Mouse Model

Four-week-old female severe combined immunodeficient (SCID) mice were purchased from the National Laboratory Animal Center (NARLabs, Taipei, Taiwan). Ethical approval of the animal care and experimental procedures was obtained from the Taipei Medical University Laboratory Animal Center (IACUC approval No: LAC-2014-0282). PF127, a nonionic triblock copolymer, namely, poly (ethylene oxide)-poly (propylene oxide)-poly (ethylene oxide) (PEO-PPO-PEO), developed from the self-assembly of two monomeric units, ethylene oxide, and propylene oxide, in water can show amphiphilic characteristics in aqueous environments. HA (0.1 and 1%) was premixed with PF127 solution at room temperature (25 °C). The HA/PF127 solution was then mixed with 5 × 10^6^ MDA-MB-231 cells and transplanted into SCID mice, in which the HA/PF127/cell matrix mixture changed to a hydrogel at 37 °C due to the temperature-sensitive characteristic of PF127 [16]. To image tumor cells that stably express luciferase, the mice were intraperitoneally injected with 100 μL of luciferin solution. Ten minutes after the luciferin injection, the mice under anesthesia using inhalation of isoflurane were imaged under an IVIS 200 (Xenogen, Alameda, CA, USA) bioluminescence imaging system quantitatively measure tumor bioluminescence. During the experiment, the tumor size was measured using calipers, and the tumor volume was estimated by using the following formula: tumor volume (mm^3^) = 1/2 × L × W^2^, where L was the length of the tumor and W was the width of the tumor.

### 2.19. In Vivo Breast Cancer Allograft C57B6/J-Has3-KO Mouse Model

Four-week-old female C57B6/J mice were purchased from the National Laboratory Animal Center (NARLabs, Taipei, Taiwan). Ethical approval for animal care and experimental procedures was obtained from the Taipei Medical University Laboratory Animal Center (IACUC approval No: LAC-2016-0317). E0771 murine breast cancer cells (5 × 10^6^) were subcutaneously inoculated into the backs of mice (wild-type C57B6/J mice and C57B6/J-Has3-KO mice). The mice were imaged for quantitative measurement of tumor bioluminescence intensity, as described above.

### 2.20. Ex Vivo Tumors: Isolation and Processing

The primary cultured lung-metastatic E0771 breast tumor cells (E0771-M) were harvested from the mouse E0771-allografted tumor. Tumor tissues were initially subjected to mechanical breakdown by mincing the tumors with a scalpel, followed by chemical digestion in 10 mL of 0.2% collagenase A (Sigma Aldrich 10103578001, Saint Louis, MO, USA) and 0.2% trypsin (Gibco 27250018, Amarillo, TX, USA), and 0.5% FBS in RPMI for 1 h at 37 °C. After digestion, tumor cells were cultured in Dulbecco’s Modified Eagle Medium/Nutrient Mixture F-12 (DMEM/F12, Gibco, Amarillo, TX, USA), supplemented with 10% heat-inactivated fetal bovine serum (FBS; Gibco, Amarillo, TX, USA) and 50 U/mL penicillin/streptomycin/neomycin (Invitrogen, Waltham, MA, USA), in a humidified (5% CO_2_, 37 °C) incubator.

### 2.21. In Vivo Breast Cancer Patient-Derived TNBC Tumor Xenograft Mouse Model

Patients enrolled in the study were selected based on basal-like triple negative breast cancer from the TMU biobank (TMU-IRB, P102025, Taipei, Taiwan). Non-obese diabetic–severe combined immunodeficiency–IL2R gamma null (NSG) mice were obtained from breeding pairs originally purchased (JAX#4659679) from Jackson Laboratories (005557, Bar Harbor, ME, USA). NSG mice were bred in a pathogen-free unit and maintained in sterile cages. Mice were handled and cared for with strict adherence to guidelines established by the Animal Resource Center, and following study protocols as approved by the Laboratory Animal Center and Use Committee at Taipei Medical University (IACUC protocol LAC-2018-0343). Breast tumor biopsy specimens from six cases were transplanted into the fat pads of female NSG mice according to standard protocols [17,18]. No enzymatic or mechanical tumor dissociation was performed at the first passage in mice. The animals were monitored for engraftment by routine palpation, and the tumors were harvested when they reached a volume of 0.8 cm^3^. The same protocol was applied to further passages in mice up to the third generation (F3) of transplantations. The tumors in each case were cut into six pieces and transplanted into immunodeficient NSG mice at the fourth mammary gland (area B, one piece per mouse, for a total of three mice) and the area distal to the mammary gland (area A, one piece per mouse for a total of three mice) of female NSG mice in different mice. Tumor tissues were monitored once a week. After 69 days, tumor-associated parenchymal tissues were harvested for additional experiments [19].

### 2.22. Fluorescent Immunohistochemistry (IHC) Staining

CD44 and HA protein interactions in PDX and human breast tumor tissues were detected by fluorescent IHC staining. Paraffin-embedded PDX tissues were cut into 8-μm sections. The sections were preincubated in 3% H_2_O_2_ and 0.3% Triton X-100 before microwaving for antigen retrieval. For CD44 and HA immunostaining, sections were microwaved in Tris buffer (pH 6) for 10 min. For HA antibody (GTX17370, GeneTex, Irvine, CA, USA) blocking by HA (96144, Sigma-Aldrich, Saint Louis, MO, USA), the HA antibody was blocked by 1% of HA for 2 h. Following this step, the antigenicity of the tumor cells in sections was blocked in 5% horse serum (Chemicon, Temecula, CA, USA) for 30 min and subsequently incubated with diluted (1:100) CD44 (GTX628472, GeneTex, Irvine, CA, USA)- and HA (GTX17370, GeneTex, Irvine, CA, USA)-specific antibodies for 2 h at room temperature. AffiniPure goat anti-mouse rhodamine (200-022-037, Jackson ImmunoResearch, West Grove, PA, USA) and AffiniPure goat anti-rabbit FITC (111-095-003, Jackson ImmunoResearch, West Grove, PA, USA) secondary antibodies were diluted 1:50. Slides were incubated with secondary antibodies for 1 h at room temperature. After secondary antibody incubation, slides were immersed in Autofluorescence Eliminator Reagent (3016635, Sigma-Aldrich, Saint Louis, MO, USA) for 5 min. Slides were mounted with VECTASHIELD Antifade Mounting Medium (H-1000, Vector Laboratories, Burlingame, CA, USA) and imaged via confocal microscopy (DMI 6000B CS, Leica, Wetzlar, Germany).

### 2.23. Statistical Analysis

All data are expressed as the means with 95% confidence intervals (CIs) of at least three determinations, unless stated otherwise. A paired t-test was performed to compare HAS3 mRNA expression in paired normal tissues vs. tumor tissues dissected from breast cancer patients. The difference in HAS3 mRNA expression detected in tumor samples vs. normal samples was analyzed using the Scheffe test. All statistical comparisons were performed using Sigma Plot graphing software (Version 10, San Jose, CA, USA) and IBM SPSS Statistics, Version 21.0 (Armonk, NY, USA: IBM Corp.). All statistical tests were two-sided. A *p* value of 0.05 or less was considered to indicate statistical significance.

## 3. Results

### 3.1. Higher HAS3 Protein Expression was Detected in the Stromal ECM of Breast Tumor Tissue

**Figure 1:**
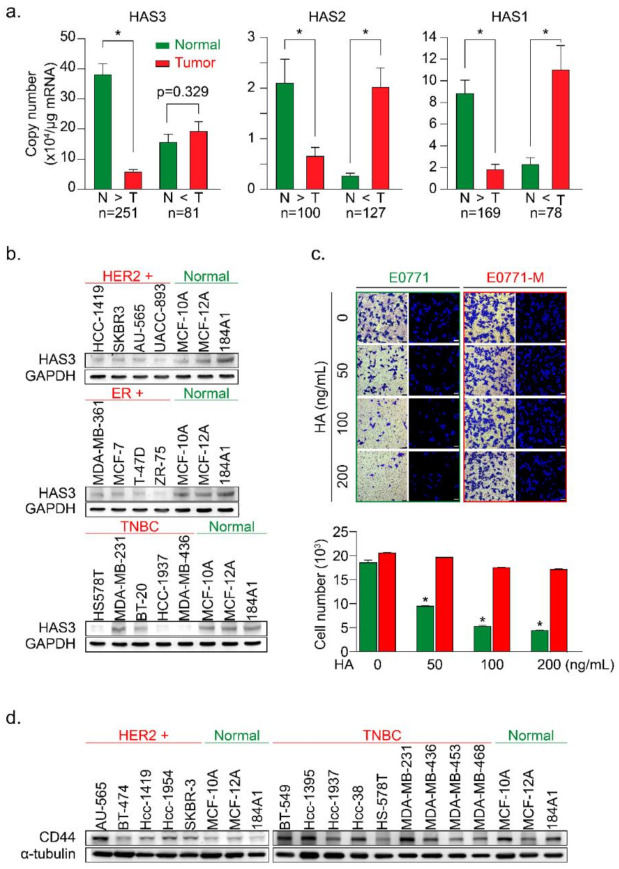
The HAS3 protein was preferentially overexpressed in normal breast epithelial cell lines and adjacent normal tissues surrounding the breast tumor. (**a**) The HAS family (HAS1, 2, and 3) mRNA expression profiles of paired human breast tumor (red bars) and normal (green bars) tissues were determined by real-time PCR. Copy numbers (× 10^4^ per μg of mRNA) were calculated from mean real-time PCR data; error bars indicate 95% confidence intervals. Data were analyzed with a paired t-test, and the presented *p*-values are two-sided. Normal tissue (N) vs. tumor tissue (T) in each group. * *p* < 0.0001; (**b**) The protein expression of HAS3 in 13 breast cancer and three normal breast epithelial cell lines was detected by immunoblotting. The protein expression of GAPDH is shown as a protein loading control; (**c**) A Transwell assay was conducted to test the HA-induced anti-migratory effects. HA (0–200 ng/mL) was added in the lower chamber coculture with the syngeneic mouse breast cancer (E0771, green bars) and lung-metastatic E0771 (E0771-M, red bars) cancer cells in the upper chamber. The data shown indicate the number of migrated mouse (E0771 vs. E0771-M) breast cancer cells. Error bars indicate 95% confidence intervals. Data were analyzed with a paired t-test, and the presented *p*-values are two-sided. * *p <* 0.05 compared with the control group; Magnification, 630×; scale bar, 200 μm. (**d**) The protein expression of CD44 in 14 breast cancer cell lines and three normal breast epithelial cell lines was detected by immunoblotting. The protein expression of α-tubulin is shown as a protein loading control.

In this study, we examined the mRNA expression of the HAS family members (HAS1, 2, and 3) in paired tumor (T) vs. normal (N) tissue samples by real-time PCR analysis (Figure 1a). HAS1, HAS2, and HAS3 mRNA expression levels were evaluated for correlations with clinical parameters (Table 1, Table 2 and Table 3), and the results were calculated and divided into two groups (N > T and T > N). We found that the mRNA expression of HAS3 was detected at a higher level (22-fold) significantly in the normal tissues of paired tumor samples within the N > T group (Figure 1a, *p* < 0.0001). However, the HAS1 and HAS2 mRNA expressions were detected and had similar expression profiles in both (N > T and T > N) groups (Figure 1a). Among the N > T group, 10-fold higher HAS3 mRNA expression was detected preferentially (142/250, 56.8%) in normal tissues compared with tumor tissues of paired samples (Supplementary Figure S1a, blue bar 4). However, in the T > N group, HAS3 mRNA expression in most tumor tissues was less than 10-fold than in the paired normal tissues (Supplementary Figure S1a, yellow bars 1–3).

We then used breast cancer (*n* = 13) and normal epithelial (*n* = 3) cell lines to test this hypothesis. The protein expression levels of HAS3 were detected by Western blot analysis (Figure 1b). Interestingly, we found that the HAS3 protein was seen at a higher level in normal breast epithelial cells (MCF-10A, MCF-12A, and 184A1) when compared to the breast cancer cell lines (Figure 1b). The results of IHC experiments in breast cancer tissue sections also revealed that HAS3 protein expression was significantly higher in normal tissue (green rectangle) than in tumor tissue (red rectangle) (Supplementary Figure S1b).

These results suggest that HAS3 or its enzymatic product (hyaluronan, HA), one of the principal constituents in normal breast stromal tissues near the tumor area, may have an essential role in attenuating tumor cell invasion. To mimic this condition in vitro, a Transwell assay in which various MW length of HA (50–200 ng/mL) contained in the lower chamber was added, and the migratory activity of breast cancer (MDA-MB-231) cells cultured in the upper chamber was evaluated (Supplementary Figure S1c). The results revealed that the addition of HA (>50 ng/mL) with an MW range from 8 to 750 kDa to the lower chamber effectively inhibited the migration of human breast cancer cells (MDA-MB-231) (Supplementary Figure S1c, * *p* < 0.0038).

Previous papers have indicated that highly migratory breast cancer cells express higher levels of CD44 antigen, which is a cell-surface glycoprotein involved in tumor cell migration, and is a receptor for HA [20]. This result is inconsistent with our work as described above. We hypothesize that HA within the ECM of breast stroma did not provide enough inhibition effects for cell migration to the highly migratory breast cancer cells. We established two primary cultured cell lines from mouse breast cancer cells (E0771) xenograft tumor with luciferase activity to test this possibility. The primary cultures of tumor cells from the original tumor (red arrow, named E0771) and lung metastasis (yellow arrow, named E0771-M) sites were established (Figure 1c and Supplementary Figure S1d). The cells were cultured in various HA concentrations in the Transwell assay analysis described above (Figure 1c). Surprisingly, we found that the lung-metastatic E0771-M cancer cells were resistant to the migration inhibition effects of HA (70–120 kDa) in the Transwell assay (Figure 1c, red bars, * *p* < 0.001).

A previous study demonstrated the putative role of CD44 in HA-mediated carcinogenic signaling [21,22]. We then analyzed the protein expression of CD44 in various types of human breast cancer cell lines (Figure 1d). The results indicated that CD44 was detected in TNBC cells, such as MDA-MB-231, derived from a metastatic site of human breast cancer compared to the HER2+ breast cancer cells, except for the AU-565 cell line, which was isolated from a metastatic site of human breast cancer [23]. These results implied that the highly metastatic cancer cells (such as E0771-M) were resistant to HA-induced migration inhibition effects (Figure 1c). We suggested that HA attracts the highly migratory cancer cells (E0771-M) at the margin of the original tumor site to move into the HA-rich stromal area of tumor tissue. An in vivo study was conducted to evaluate the content of stroma HA in normal stroma tissues compared to the tumor-derived HA in patient-derived xenografted (PDX) tumors from NSG mice (Supplementary Figure S1e). The results show that compared with tumor tissues, the concentration of HA detected in the matrix is higher (Supplementary Figure S1e).

We hypothesized that HA detected in the tumor tissues was synthesized by human breast tumor-derived fibroblasts (HB-TDFs), which surround the original cancer lesions, may suppress cancer cell migration. To test this hypothesis, primary HB-TDFs (named F-1 to F-3) established from the ECM of human breast tumor tissues were cultured for 24 h, and the medium supernatant was collected. The concentration of HA synthesized by the HB-TDFs was determined in the supernatant (denoted as a conditioned medium) by ELISA (Supplementary Figure S1f). The HAS3 protein expression level was higher in the F-3 cells than in the other (F-1 and F-2) HB-TDFs. As expected, the HA concentration was highest in the conditioned medium from F-3 HB-TDFs (Supplementary Figure S1f, * *p <* 0.028). In order to test whether HA derived from HB-TDF in the conditioned medium has an inhibitory effect on tumor cell migration, two representatives HB-TDF (F-2 and F-3, lower chamber) were selected and combined with MDA-MB-231 co-cultivation in the Transwell plate (upper chamber) (Supplementary Figure S1g). The data indicated that the conditioned medium from F-3 HB-TDFs cells secreting higher HA levels which inhibited MDA-MB-231 cell migration than the F-2 derived HA-treated group (Supplementary Figure S1g, * *p <* 0.012). This finding suggests that HB-TDFs cells produced HA in the ECM within breast tumor tissues, having an anti-migratory effect. Furthermore, migratory cancer cells (such as E0771-M) were resistant to the anti-migratory effect of HA (Figure 1c) by some unknown mechanisms.

### 3.2. HAS3 Deficiency in Normal Stroma Tissue Promoted Breast Cancer Tumorigenesis

**Figure 2:**
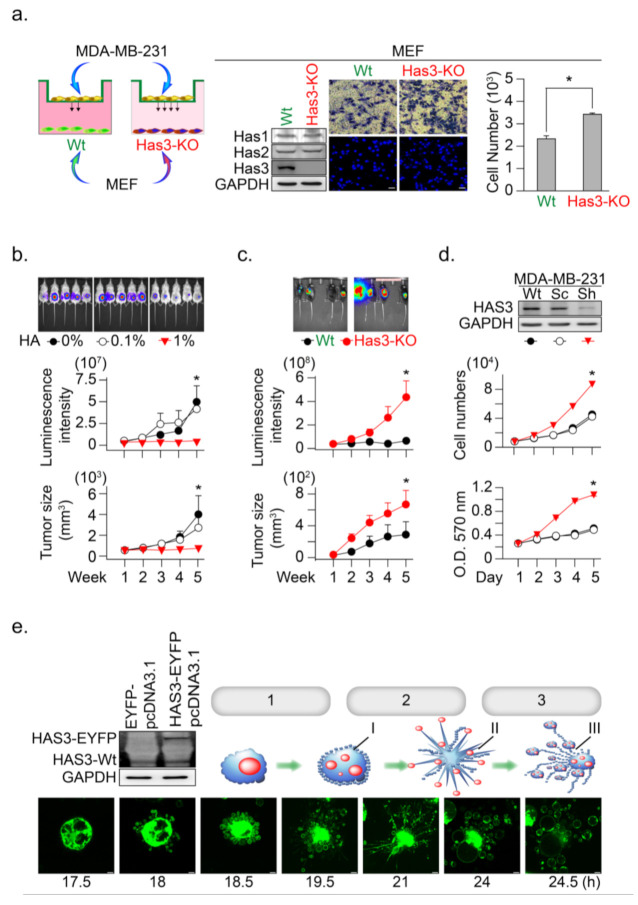
HAS3 deficiency in normal stroma tissue promoted breast cancer tumorigenesis. (**a**) A Transwell assay was conducted to test the anti-migratory effects of condition medium from primary cultured mouse MEF. The MEF derived from wild type and HAS3 KO mice were seed in the lower chamber coculture with the MDA-MB-231 cancer cells in the upper chamber for 24 h. The data shown indicate the number of migrated breast cancer cells. Error bars indicate 95% confidence intervals. The data are presented as the mean ± SD. Data were analyzed with a paired t-test, and the presented *p*-values are two-sided. * *p* *=* 0.00061 compared with the control group. We performed western blot analysis, and the results indicated that HAS3 KO did not affect the HAS2 and HAS1 protein levels in both MEF cells. Magnification, 200×; scale bar, 50 μm; (**b**) In vivo HA exposure inhibited MDA-MB-231 cell xenograft tumor growth in mice. Tumor growth was monitored by measuring tumor volume with the IVIS200 system weekly until five weeks after cell transplantation. The data are presented as the mean ± SD; * *p* *<* 0.05 compared with the control group; (**c**) E0771 cells were transplanted subcutaneously into the backs of wild-type (C57B6/J) and C57B6/J-Has3-KO mice (*n* = 5). Tumor growth was monitored by measuring tumor volume with the IVIS200 system every week until five weeks after cell transplantation. The data are presented as the mean ± SD; * *p* *<* 0.05 compared with the control group; (**d**) Knockdown of HAS3 by the pSUPER-Sh-HAS3 plasmid transfection in MDA-MB-231 cells promoted cell proliferation. Cell proliferation was increased in HAS3 pSUPER-Sh-HAS3 plasmid-transfected MDA-MB-231 cells, as determined by MTT assay or cell quantification. The HAS3 protein level was detected by Western blot analysis. The GAPDH protein level was detected as a control to ensure equal protein loading (left panel). The data are presented as the mean ± SD; * *p* < 0.05 compared with the control group; (**e**) Live-cell time-lapse confocal images show changes in the gross morphology of MDA-MB-231 cells transfected with HAS3-overexpression (HAS3-EYFP-pcDNA3.1) plasmid. Different steps of apoptotic cell death were denoted as 1. apoptotic membrane blebbing, 2. formation of apoptotic membrane protrusions, 3. cell fragmentation, I. small surface membrane blebs, II. apoptopodia, and III. apoptotic bodies. Representative time-dependent data were selected. Magnification, 630×; scale bar, two μm.

The above results indicated the anti-migratory effects of HA produced from HB-TDFs cells in the tumor tissue; we then tested whether a scanty amount of HA in normal stroma tissue promotes tumor cell invasion. We established embryonic fibroblasts from HAS3 knockout C57B6/J-Has3-KO mice (named MEFs-Has3-KO) that produced low concentrations of HA (Figure 2a). The cells (wild-type MEFs and MEFs-Has3-KO) were seeded onto the lower chambers of the Transwell plate for 24 h, and the migratory activity of the MDA-MB-231 cell was determined (Figure 2a). The results indicated that lower HA concentrations in the conditioned medium derived from MEF-Has3-KO cells significantly increased MDA-MB-231 cell migration to the membrane compared to wild-type MEFs (Figure 2a, * *p =* 0.00061). This result suggested that a scanty amount of HA in normal stroma tissue promotes the initial stage of tumor cell invasion.

Such results proposed that higher HA in tumor tissue may have an inhibitory effect to reduce tumor cell growth. An in vivo study was conducted to mimic a microenvironment with higher HA concentration in the ECM of xenograft tumor-bearing mice; the tumor cells were premixed with a HA solution (0.1 and 1%, *w*/*v*) in a temperature-sensitive PF127 [16] solution at room temperature (25 °C). The cell-matrix (contained HA and PF127) was transplanted into SCID mice, in which the cell-matrix changed from liquid to a semisolid hydrogel form at body temperature (37 °C) [16]. The HA in this mixture was slowly released into the microenvironment of MDA-MB-231-xenografted tumors, after which tumor growth was determined (Figure 2b). The results indicated that HA (1%, *w*/*v*) contained in MDA-MB-231 tumor cell-matrix significantly inhibited xenograft tumor growth, as evaluated by the luminescent intensity and tumor volume in the SCID mice model (Figure 2b, * *p* *<* 0.05).

To further confirm this hypothesis, we established HAS3 gene knockout (C57B6/J-Has3-KO) mice to generate a low HA in the tumor microenvironment. Syngeneic mouse breast tumor cells (E0771) were transplanted subcutaneously into C57B6/J-Has3-KO or control wild-type C57B6/J mice. The luminescent intensity and tumor volume evaluated tumor growth according to standard methods. The growth rate of breast tumors increased significantly in mice lacking HAS3 compared with wild-type mice (Figure 2c, * *p* *<* 0.05). These results indicated that HAS3 protein depletion in C57B6/J-Has3-KO mice establishes a microenvironment lacking specific types of HA in the ECM that promote breast tumor cell growth.

The IHC staining results indicated that the HAS3 protein expression was lower in human breast tumor tissues than the adjacent normal tissues (Supplementary Figure S1b). Such results suggested that the HAS3 protein expression in tumor cells directly inhibits tumor cell growth. We established a stabled HAS3 protein inhibiting MDA-MB-231 cells by the shRNA technique, in which the protein expressions of HAS1 and HAS2 were not affected, and cell growth proliferation was determined (Figure 2d). The inhibition of HAS3 protein expression significantly increased tumor growth than the scrambled control sequence treated cells (Figure 2d). To test the opposite phenomena observed in MDAMB231 cells, we further test whether overexpression of the HAS3 protein inhibits tumor cell growth. The HAS3 protein was forced-overexpressed in the MDA-MB-231 cells, and continuous confocal time-lapse images observed the HAS3-induced cell death effects. As shown in Figure 2e, HAS3 overexpression in cancer cells promoted a series of apoptotic processes, including membrane blebbing (denoted as step-1), the formation of apoptotic membrane protrusions (indicated as step-2), and cell fragmentation (marked as step-3). These results can explain the pathological observation of lower HAS3 protein expression in breast tumor tissue (Supplementary Figure S1b).

### 3.3. HAS3 Overexpression in Human Breast Cancer Cells Arrests the Cancer Cell Cycle at the G2/M Phase

**Figure 3:**
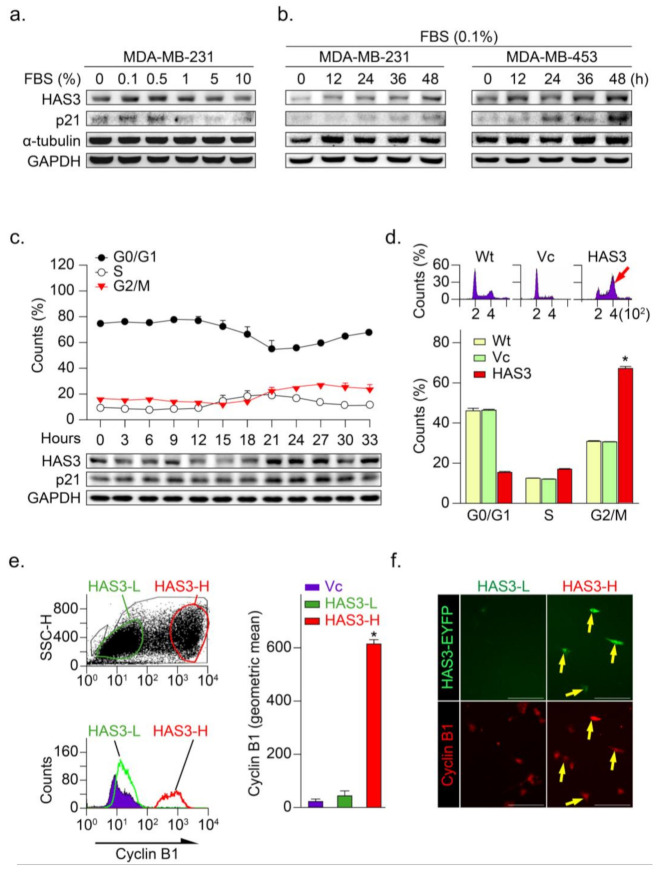
HAS3 overexpression arrested human breast cancer cells at the G2/M phase cell cycle. (**a**) MDA-MB-231 cells were treated with media containing serum at different concentrations (0–10%) for 24 h and harvested to detect the protein levels of HAS3, p21, α-tubulin, and GAPDH by Western blot analysis; (**b**) The protein levels of HAS3, p21, α-tubulin, and GAPDH were detected by Western blot analysis in breast cancer cells (MDA-MB-231 and MDA-MB-453), after being treated with 0.1% FBS for 12–48 h; (**c**) Flow cytometry analysis of MDA-MB-231 cells was performed, and the fraction of cells at G0/G1, S, and G2/M phase was assessed by PI staining (upper panel). The protein levels of HAS3, p21, and GAPDH were detected by Western blot analysis. The data are presented as the mean ± SD relative to the cell population (%); (**d**) HAS3 overexpression in MDA-MB-231 cells arrested cell proliferation at the G2/M phase of the cell cycle. Representative flow cytometry histograms showing alterations in the cell cycle of MDA-MB-231 cells 24 h after transfection. The data are presented as relative to the cell population (%). Data were analyzed with a paired t-test, and the presented *p*-values are two-sided. * *p* < 0.05 compared with the Wt group. (**e**) HAS3 overexpression was simultaneously detected in cyclin B1 protein-positive stained MDA-MB-231 cells. Flow cytometry dot plot analysis was performed in HAS3-EYFP-pcDNA3.1 plasmid- and EYFP-pcDNA3.1 plasmid-transfected cells (left, upper). The flow cytometry histograms show that cyclin B1 expression in MDA-MB-231 cells was detected by using a rhodamine-labeled antibody against cyclin B1 (left, lower). The results were quantified according to the geometric mean signal for rhodamine (cyclin B1) (right). The data are presented as the mean ± SD; * *p* *=* 0.00028 compared with the control group. HAS3-L, Has3 lower expression; HAS3-H, HAS3 higher expression; (**f**) We further detected cyclin B1 protein expression in the population of HAS3-H and HAS3-L cells by IF staining. The results indicated that cyclin B1 protein overexpression was detected preferentially in HAS3-H cells (yellow arrows). Magnification, 630×; scale bar, 200 μm.

Our previous study demonstrated that serum starvation significantly induced neuroblastoma (N2a) cancer cell differentiation through the HAS3 protein expression [10]. In this study, low serum concentration (<0.5% FBS for 24 h) significantly induced HAS3 protein expression in MDA-MB-231 cancer cells (Figure 3a). Time-dependent experiments demonstrated that treatment with 0.1% FBS as early as 12 h significantly induced HAS3 protein expression in cancer cells (Figure 3b). The p21 cell growth cycle inhibitory protein expression was upregulated in the HAS3 expressing cells, consistent with HAS3 protein expression (Figure 3a,b). To test whether HAS3 expression is involved in regulating the cell cycle in cancer cells, MDA-MB-231 cells were synchronized by treatment with 0.04% FBS for 48 h, according to our previous paper described [24]. The synchronized cells were then treated with 10% FBS to activate the cell cycle, and the cells were harvested for flow cytometry analysis (Figure 3c). The results indicated that HAS3 and p21 protein expression in cells at the G2/M phase was upregulated simultaneously, starting at 21 h after 10% FBS treatment (Figure 3c, lower panel). These results implied that serum starvation-induced HAS3 and p21 protein expression were involved in regulating the proliferation of breast cancer cells at the G2/M phase of the cell cycle.

The HAS3-induced G2/M phase cell cycle regulatory effects were explored in the MDA-MB-231 cells transfecting with the HAS3-overexpression plasmid. Interestingly, we found that the population of HAS3-overexpressing cells in the G2/M phase was significantly higher (red arrow) than the vector control-treated cells (Figure 3d, * *p* < 0.0002). These results indicated that the overexpression of HAS3 effectively induced human breast cancer cells arrested at the G2/M phase. The cells transfected by HAS3 overexpression and vector control plasmids were analyzed for flow cytometry analysis and subdivided into two populations according to the HAS3 protein expression level (low, named HAS3-L vs. high, named HAS3-H) (Figure 3e, left). The cyclin B1 expression, a marker for the G2/M phase of the cell cycle, was detected in the HAS3-H cells simultaneously (Figure 3e, red bar, * *p* < 0.0001). We further see the cyclin B1 protein expression in the population of HAS3-H and HAS3-L cells by IF staining. The cyclin B1 protein expression was detected preferentially in the HAS3-H cells (Figure 3f, yellow arrows). The results showed that overexpression of HAS3 in human breast cancer cells inhibits cell proliferation through cell cycle arrest at the G2/M phase.

### 3.4. HAS3 Induces G2/M Phase Cell Cycle Arrest in Cancer Cells through Activation of Microtubule Hyperacetylation

**Figure 4:**
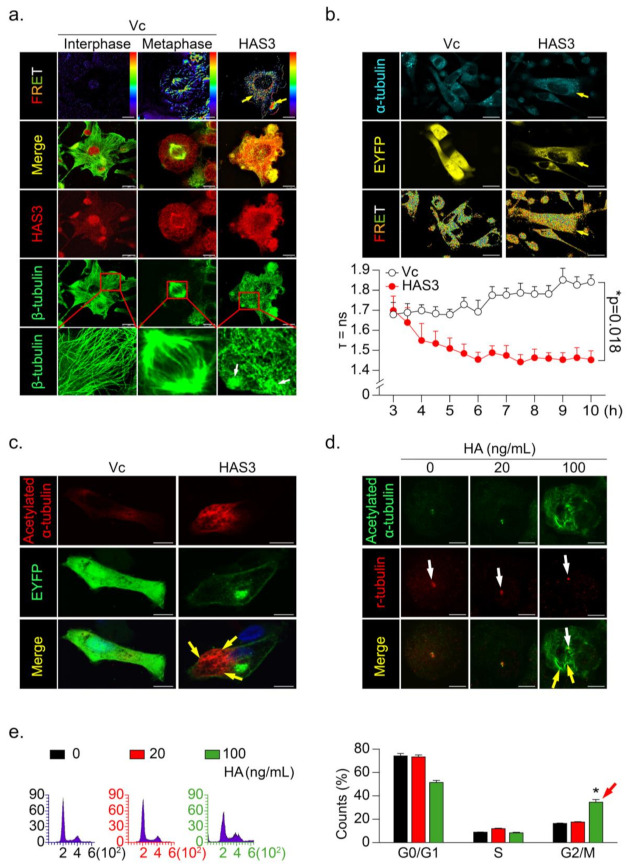
HAS3 overexpression promoted microtubule hyperacetylation through the direct binding of HAS3 to microtubules. (**a**) The interaction of β-tubulin with HAS3 in MDA-MB-231 cells was detected by FRET assay. HAS3/β-tubulin protein complex formation in cells was examined under a confocal microscope imaging system via FRET analysis. Magnification, 630×; scale bar, 7.5 μm; (**b**) Time-lapse measurement (FLIM assay) of the interaction of α-tubulin with HAS3 in MDA-MB-231 cells. FLIM occurred between the donor fluorophore (AmCyan) and acceptor fluorophore (EYFP) upon excitation of the donor fluorophore, only when the distance between fluorophores was less than 10 Å. Magnification, 630×; scale bar, 25 μm. The fluorescence lifetime decay kinetics for α-tubulin-AmCyan (FRET donor) in the presence of HAS3-EYFP-pcDNA3.1 or EYFP-pcDNA3.1 (FRET acceptor) were determined by FLIM microscopy. The mean lifetime of each emission band of AmCyan was collected every 30 min for 10 h, and the values were plotted along the wavelength (lower panel). The data are presented as the mean ± SD; * *p* = 0.018 compared with the control group; (**c**) To observe the hyperacetylation of microtubules, HAS3-EYFP-pcDNA3.1 and EYFP-pcDNA3.1 plasmid transfection in MDA-MB-231 cells was fixed and stained with anti-acetylated α-tubulin/AffiniPure goat anti-mouse-rhodamine; Magnification, 630×; scale bar, 10 μm. (**d**) HA-treated (0–100 ng/mL) MDA-MB-231 cells were fixed and stained with anti-acetylated α-tubulin/AffiniPure goat anti-mouse FITC and anti-γ-tubulin/AffiniPure goat anti-rabbit rhodamine. The protein expression of γ-tubulin in the cells is indicated by the white arrow, whereas the yellow arrow indicates acetylated α-tubulin. Magnification, 630×; scale bar, ten μm; (**e**) HA-induced G2/M cell cycle arrest in MDA-MB-231 cells. Flow cytometry was performed, and histograms show alterations in the cell cycle after 24 h of HA treatment detected by staining with PI (red arrow). Each phase of the cell cycle is presented relative to the total cell population. Data were analyzed with a paired t-test, and the presented *p*-values are two-sided. * *p* < 0.05 compared with the control group.

To further explore the mechanisms of HAS3 overexpression-induced cancer cell arrest at the G2/M phase, immunofluorescence staining was performed, and the interaction between the HAS3 and β-tubulin proteins was detected by fluorescence resonance energy transfer (FRET), according to the method described in our previous study [25] (Figure 4a). We found a significant HAS3/β-tubulin protein–protein interaction in the HAS3-overexpressing MDA-MB-231 cells compared with vector-treated control cells (Figure 4a, yellow arrows). The HAS3/β-tubulin protein–protein interaction induced abnormal polymerization in the microtubule formation (Figure 4a, white arrows). The results explain the mechanisms that HAS3-induced G2/M phase cell cycle arrest, as described above. A live-cell confocal and FRET assay was performed to confirm these observations further, and the MDA-MB-231 cells were transiently transfected with both HAS3-EYFP-pcDNA3.1 and α-tubulin-AmCyan-pcDNA3.1 plasmids. A time-dependent FLIM assay was performed, and the results showed that HAS3 interacted with α-tubulin immediately (yellow arrow, less than 4 h) after plasmid transfection (Figure 4b).

Previous studies showed that tubulin acetylation-induced microtubule dis-organization directly caused G2/M phase cell cycle arrest and apoptosis [26,27,28,29]. To test whether HAS3 indeed induced hyperacetylated α-tubulin formation in breast cancer cells, the acetylated α-tubulin-specific antibody was used for immunofluorescence staining analysis. The results demonstrated that the acetylated α-tubulin was increased in the HAS3 overexpressing MDA-MB-231 cells (Figure 4c, yellow arrow). We further tested whether the HA (20–100 ng/mL, for 24h) exposure directly induced these effects by immunofluorescence staining analysis. The acetylated α-tubulin in HA-treated MDA-MB-231 cells was detected (Figure 4d, yellow arrow), especially in mitotic cells (γ-tubulin saw as a marker) (Figure 4d, white arrow). The same experiment was performed for flow cytometry analysis, and the results indicated that HA (> 100 ng/mL) exposure significantly increased G2/M phase cell cycle arrest in MDA-MB-231 cells compared with control cells (Figure 4e, * p < 0.05). The results described above indicated that HAS3 and its enzymatic product HA in stroma ECM of breast tissues might attenuate breast cancer cell invasion by arresting early stage breast cancer (ductal carcinoma in situ, DCIS) cell growth at the G2/M phase cell cycle.

### 3.5. HAS3 Overexpression Promotes Breast Cancer Cell Autophagy

**Figure 5:**
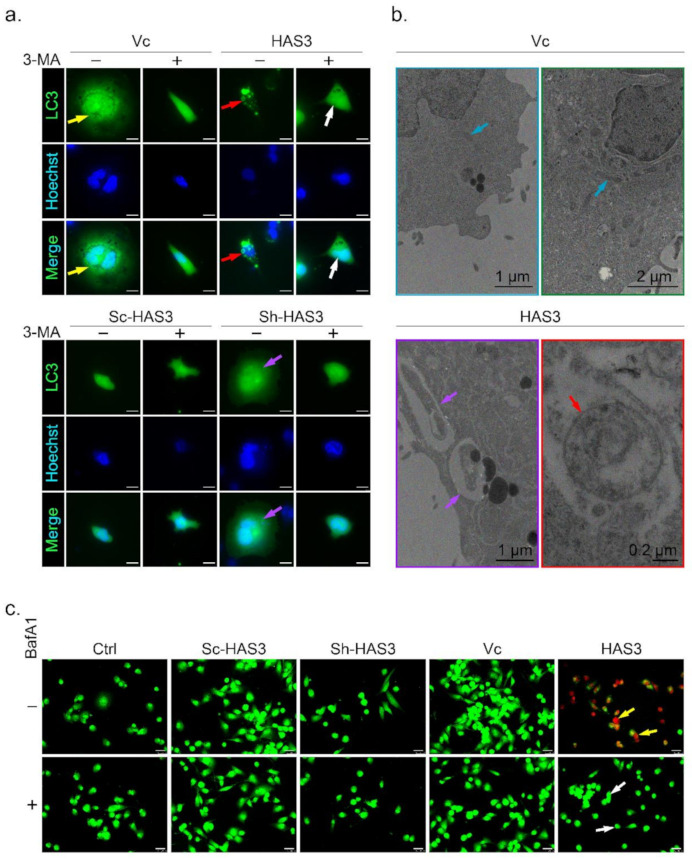
HAS3 overexpression induced autophagic cell death in breast cancer cells. (**a**) HAS3 overexpression in MDA-MB-231 cells increased LC3-GFP punctate formation. The live transfected cells were imaged by DeltaVision microscopy. A diffuse nuclear and cytosolic punctate signal for LC3-GFP, indicating autophagic cells, was detected in LC3-GFP plasmid- and HAS3-pcDNA3.1 plasmid-cotransfected MDA-MB-231 cells (red arrow), but not LC3-GFP plasmid- and pcDNA3.1 plasmid-cotransfected cells (yellow arrow). Moreover, 3-MA (0.5 mM, 24hr) pretreatment completely inhibited the LC3-GFP punctate formation indicated by the lack of autophagic cell death (white arrow). Magnification, 630×; scale bar, 25 μm; +: 3-MA treatment, -: nontreatment; (**b**) MDA-MB-231 cells were transfected with HAS3-pcDNA3.1 or pcDNA3.1 plasmid and subjected to ultrastructural analysis by TEM. MDA-MB-231 cells transfected with the vector contained typically shaped organelles (blue arrow). HAS3-pcDNA3.1-transfected cells exhibited autophagic vesicles and autolysosomes containing cytoplasmic materials (red arrow). HAS3-pcDNA3.1-transfected cells also showed various mitochondria with a disorganized structure, disrupted cristae, and swollen appearance (yellow arrow). Magnification, 12000×; scale bar, one μm; (**c**) MDA-MB-231 cells were transiently transfected with HAS3-pcDNA3.1 (HAS3), pcDNA3.1 (Vc), pSUPER-Sh-HAS3, and pSUPER-Sc-HAS3 plasmids for 24 h, and autophagic cells were evaluated by the AO staining method (yellow arrow). HAS3-induced AO staining was decreased when cells were pretreated with BafA1 (50 nM, for 24 h). BafA1 pretreatment completely inhibited the acidification of vacuoles indicated by the lack of red fluorescence (white arrow). Magnification, 630×; scale bar, 25 μm. +: BafA1 treatment, -: nontreatment.

A previous study demonstrated that microtubule hyperacetylation promotes autophagy in human cancer cells [30]. We then test whether the HAS-mediated antitumor effects induced autophagic cell death is the primary mechanism. One of the essential characters for autophagic cell death is the formation of the autophagosome, a spherical structure with a double-layered membrane. The LC3 can be used as a marker to identify autophagosomes by immunocytochemical detection. In this study, MDA-MB-231 cells were transfected with the LC3-GFP plasmid to generate an in vitro cell research model (named LC3-GFP-MDA-MB-231) used to detect autophagosome formation [31] (Figure 5a, indicated by yellow arrow). The results showed that the LC3-labeled autophagosomes were increased in the HAS3 overexpression cells (Figure 5a, red arrow). The phenomenon was attenuated by treatment with 3-methyladenine (3-MA), an inhibitor to study the mechanism of autophagy (lysosomal self-degradation) and apoptosis under various conditions [32] (Figure 5a, white arrow). The HAS3 inhibition by shRNA attenuates the autophagosome formation compared to the scrambled sequence vector control group in the LC3-GFP-MDA-MB-231 cells (Figure 5a, purple arrow). 

The autophagosome accumulation and the gross morphology changes were seen by TEM (Figure 5b). The TEM data revealed that the double-membraned autophagosomes (red arrow) with the mitochondria grossly distorted (purple arrow) were observed in the HAS3 overexpressing plasmid-transfected MDA-MB-231 cells (Figure 5b). MDA-MB-231 cells transfected with the pcDNA3.1 plasmid were used as a control group, and normal mitochondrial morphology was detected (Figure 5b, blue arrows). The acidic vacuoles (lysosomes, endosomes, and autophagosomes) that appear in the cell can be stained by acridine orange (AO) dye promising to identify autophagic death [33]. The results demonstrated that HAS3 overexpression significantly increased the number of AO-stained cells (Figure 5c, yellow arrow). BafA1, a macrolide antibiotic isolated from *Streptomyces* species, is a specific inhibitor of autophagy [34]. The HAS3-induced acidification of vacuoles was attenuated entirely by pretreatment of BafA1 (50 nM, 24 h), and the red fluorescence was attenuated (Figure 5c, white arrow).

The HAS3 overexpressing MDA-MB-231 cells were used to study the underlying mechanisms of the HAS3-induced autophagic cell death. The protein lysates were harvested from cells transfected with the HAS3 overexpression plasmid for 3, 6, and 24 h, and subjected to Western blot analysis. As shown in Supplementary Figure S2a, the autophagy-related proteins, including autophagy-related 5 (ATG5), and LC3-II, were significantly upregulated in the HAS3 overexpression cells compared to the vector control group (Supplementary Figure S2a). Similar to the treatment in Figure 5c, HAS3 overexpression-induced LC3-II protein expression was increased by pretreatment with BafA1 (Supplementary Figure S2b). LC3-II protein upregulation was not observed in the pSUPER-Sh-HAS3 plasmid transfected cells. All these results demonstrated that HAS3 overexpression significantly induced autophagic cell death in MDA-MB-231 cells.

### 3.6. HAS3 Expression in Cutaneous Tissue Inhibits TNBC-PDX Tumor Growth in Immunodeficient Mice

**Figure 6:**
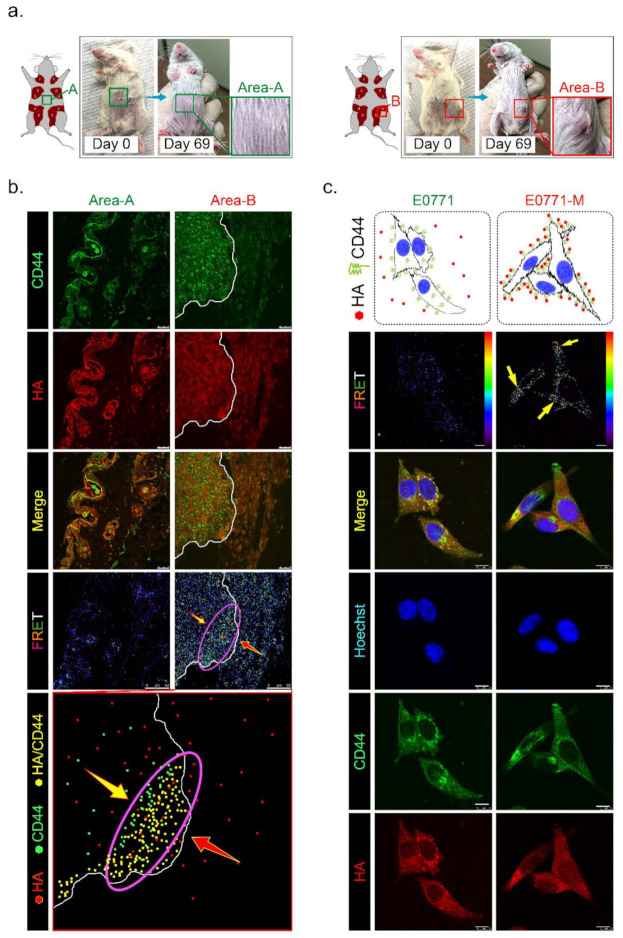
Has3 expression in the parenchymal tissues inhibited TNBC-PDX tumor growth in immunodeficient (NOD SCID gamma mouse, NSG) mice. (**a**) PDX (F3) mice were generated from 6 TNBC biopsies. Tumor tissues from the same patient were transplanted into the same mice in different areas (A and B, as indicated in the left panel); (**b**) The PDX tumor sections (area A and area B) were adapted for immunofluorescence staining by using antibodies specific to CD44 (labeled by a secondary antibody with FITC, green fluorescence) and HA (labeled by a secondary antibody with rhodamine, red fluorescence). CD44/HA protein complex formation in both area A and area B was examined under a confocal microscope imaging system via FRET analysis. The red/blue color spectrum indicates the intensity of FRET activity. Cells stained with CD44-specific antibody. Scale bar = 25 µm; (**c**) Highly metastatic mouse breast cancer (E0771-M) cells with membranous CD44 antigen were attracted by interacting with HA in vitro. The cells (E0771 and E0771-M) were cultured in a medium with 50–200 ng/mL HA for 24 h. After treatment, the cells were adapted for immunofluorescence staining using antibodies specific to CD44 (labeled by a secondary antibody with FITC, green fluorescence) and HA (labeled by a secondary antibody with rhodamine, red fluorescence). CD44/HA protein complex formation in cells was examined under a confocal microscope imaging system via FRET analysis. The red/blue color spectrum indicates the intensity of FRET activity. Scale bar = 20 µm.

To test whether HAS3 encompassed in cutaneous tissue directly influences tumor growth, we established TNBC-PDX (F4, *n* = 6) mouse models. PDX models of cancer were developed by implanting tissues from a patient’s tumor into immunodeficient mice. The PDX model was used to create an environment that allows tumor growth to be monitored. Tumor pieces from the represented cases were implanted into the fat pad area near the mammary gland tissue in mice (Figure 6a, indicated as area-B). Other tumor pieces derived from the same cases were transplanted into a region distal to the fat pad in other mice (Figure 6a, indicated as area-A). We found that the TNBC-PDX tumors derived from all cases were established in area-B, but not area-A, after 69 days of transplantation (Figure 6a, red rectangle).

Previous studies demonstrated that CD44 is a receptor for HA expressed in highly metastatic tumor cells [20]. According to Figure 1c, we found that lung-metastatic breast cancer cells (E0771-M) were resistant to the migration inhibition effects of HA in the Transwell assay. We hypothesized that highly migratory cancer cells (such as E0771-M) with functional membranous CD44 are selected from the original tumor site to invade the HA-rich stromal area of tumor tissues. The CD44 interacted with HA in the tumor/normal junction of PDX tumor tissues was determined by FRET assay (Figure 6b). The tissues from area-A (stroma alone) and area-B (tumor/stroma junction) were dissected from the PDX tumor-bearing mice, and the tissue sections were stained with antibodies specific to CD44 (labeled with FITC) and HA (tagged with rhodamine). The results indicated that the highly migratory breast cancer cells invaded from the tumor center into the margin of stromal tissues (Figure 6b, yellow arrow). In contrast, HA was synthesized and released from normal stromal tissues into the extracellular space of tumor tissues (Figure 6b, red arrow). Highly migratory breast cancer cells with CD44 expression bound to HA released from stromal tissues were detected as FRET-positive stained cells, showing that they accumulated mainly near the margins of tumor tissue (Figure 6b, purple oval circle). These results suggest that HA does not directly inhibit highly malignant cell migration through a CD44-mediated mechanism.

We proposed that HA selected highly migratory breast cancer cells which express different levels of CD44 located in tumor/stroma junction. The protein level of membranous CD44 derived from original (E0771) and lung-metastatic (E0771-M) sites was determined by immunoblotting analysis, and the results revealed that the CD44 protein level in both cell lines was similar (Supplementary Figure S3a). We then ask whether the binding affinity of CD44 and HA was different in both cancer cells. The E0771 and E0771-M cells were exposed to HA (24 h), and an immunofluorescence staining experiment was performed using CD44- and HA-specific antibodies. The FRET assay detected the membranous-bond CD44/HA interactions. The results indicated that CD44 appeared on the E0771-M cell membrane exposed to the HA-rich medium, and was tightly bound to HA with FRET activity (Figure 6c, yellow arrow). Such effects will permit the invasion and selection of highly metastatic cancer (E0771-M).

In conclusion, the HAS3 and its enzyme products were detected in the stroma breast tissues at a high level, attenuating effects for induced breast cancer cell death and inhibiting the cancer cells invasion at the initial stage. However, the highly migratory cancer cells were resistant to the HA-mediated effects with unknown mechanisms. The cells that express CD44 were selected and bound to HA in the tumor/stroma junction, and the tumor cells invasion at the initial stage. Understanding the mechanisms of CD44/HA interaction and activating the HA-induced antitumor effects in this population of cancer cells will be valuable when it comes to comprehending the stepwise mechanisms of the invasion in highly migratory tumor cells.

## 4. Discussion

The tumor microenvironment is a crucial factor in determining the aggressiveness of cancer cells [35,36,37]. Glycosaminoglycan HA is one of the principal constituents of the stromal tissue around the tumor margin. Higher HA levels in breast ECM tissue were shown to significantly improve relapse-free survival in a group of breast cancer patients (*n* = 813) with ductal tumors (*p* = 0.01) [38]. HA levels in tumor tissues were observed to have different consequences for the clinical outcome, and the MW of HA was shown to be critical. A previous study [39] demonstrated that high-MW HA (HMW-HA) binds CD44+ tumor cells, inhibiting proliferation. In contrast, low-MW HA (LMW-HA) forms a complex cluster that binds CD44+ tumor cells and promotes cell proliferation. Most studies support the idea that HMW-HA accumulation in stromal tissues is responsible for the tumor-suppressive effects of HA in cancers [8,9].

To define the MW of HA derived specifically from HAS3, the conditioned medium from MEF and MEF-Has3-KO cells was harvested. The samples with different MW cutoffs were concentrated according to the standard protocol ultracentrifugal filters sandwich ELISA-like assay (Supplementary Figure S3b) [20]. The HA concentrations in both samples from MEF and MEF-Has3-KO cells were similar, except that the conditioned medium fraction harvested from the MEF-Has3-KO cells with an MW range of 50~100 kDa was depleted (Supplementary Figure S3c). These results imply that HAS3-derived HA with a specific MW range (50~100 kDa) in the tumor tissue microenvironment plays a critical role in inhibiting cancer cell migration. HA may have paracrine effects when originating from the stromal cells and may inhibit tumor cell invasion. An in vitro study demonstrated that HA oligosaccharides exert their antitumor effects by suppressing cell growth and inhibiting migration and invasion in breast cancer cells [9]. Another study also revealed that HA accumulation was detected in the tumor stroma and cell-cell junction in an in vivo study evidenced by IHC staining [40]. Similarly, we demonstrate that the HA-induced anti-migratory effects rely on the specific MW range derived from the HAS family (Supplementary Figure S1c).

**Figure 7:**
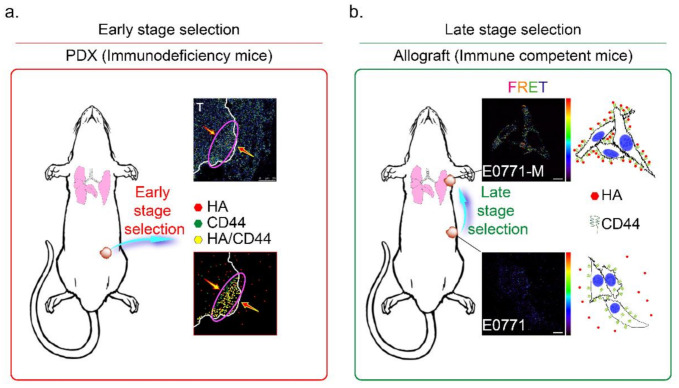
Mechanisms of HA and CD44 mediated selection of highly metastatic breast cancer cells. The metastatic tumor cells are subdivided into two populations, namely (**a**) early- and (**b**) late stage selected tumor cells. (**a**) In the early stage selection, the highly metastatic tumor cells were enriched and toward the tumor/normal stroma margin (red arrow). Highly malignant tumor cells with CD44 expression were determined by HA, which appears in the normal stroma in the early stage invasion (red oval circle); Magnification, 630×; scale bar, 50 μm. (**b**) After the first step invasion, the cancer cells invaded into blood vessels, and step to the late stage selection, the HA content of the metastatic site (lung) selectively attracts the more motile E0771-M (CD44+) breast cancer cells. Magnification, 630×; scale bar, 10 μm.

The CD44 is an essential molecular marker of cancer stem cells in breast cancer. The CD44 is expressed on the surface of highly metastatic breast cancer cells as a co-receptor for a broad diversity of extracellular matrix ligands, primarily HA [41]. Recently, a paper demonstrated that the interaction of HA with CD44 activates AMPK and autophagic pathways [42]. To explore the mechanisms of CD44-mediated breast cancer cell invasion detected in the HA-rich stroma tissues, we proposed the HA-induced metastatic cancer cell selection hypothesis in Figure 7. We suggest that the metastatic tumor cells should be subdivided into two populations, i.e., early- and late stage-selected tumor cells. In the early stage selection, highly malignant tumor cells with CD44 expression were determined by HA, which appears in the normal stroma in the early stage invasion. The HA-selected tumor cells were enriched and toward the tumor/normal stroma margin (indicated by a purple oval circle in Figure 7a). After the first step invasion, the cancer cells invaded blood vessels, and after the late stage selection, the HA content of the metastatic site (such as the lung) selectively attracts the more motile CD44+ breast cancer cells (such as E0771-M cells, Supplementary Figure S1d and Figure 6c). All these CD44+ cancer cells were resistant to HA-induced anti-migratory effects, as described above (Figure 1c). The results demonstrated that HA effectively selected the E0771-M cells established from lung tumor tissue in immune-competent mice, evidenced by increased FRET activity than the E0771 cells (indicated by yellow arrows in Figure 7b).

In this study, we investigated this process, and found that highly migratory breast cancer cells express higher levels of the CD44 antigen, which is a cell-surface glycoprotein involved in tumor cell migration (44). In addition, hyaluronic acid (hyaluronan, HA) is an anionic, nonsulfated glycosaminoglycan synthesized and distributed within connective, epithelial, and neural tissues (2). Like these pro-inflammatory cytokines (TNF-α, IL-6, and IFN-γ), HA is another principal constituent in the stromal tissues surrounding tumor tissues. The CD44 expressed in breast tumor cells is a receptor for HA, and is attracted by HA in the tumor microenvironment during invasion of tumor cells (3). For these reasons, we suggested that HA derived from the stromal tissues surrounding tumor cells should be considered as another constituent that exerts chemoattractant functions.

In this study, we detected the hyperacetylation of α-tubulin in human breast cancer cells (MDA-MB-231) by treating a culture medium containing HA or the forced expression of HAS3 by plasmid transfection (Figure 4c). Hyperacetylation of α-tubulin was also detected in human cancer cells after exposure to anticancer drugs, resulting in increased apoptosis by arresting the cell cycle in the G2/M [26] or G0/G1 phase [27,28]. Microtubule hyperacetylation also plays other roles in promoting the autophagy processes [43]. HA-induced autophagy was observed in HK-2 cells by TEM images analysis and calculated by the quantification of the levels of autophagic vacuoles [44]. There are plenty of mechanisms that can cause hyper-acetylation of alpha-tubulin. Until now, the mechanism of HA affects hyper-acetylation of alpha-tubulin is still unknown. The acetylated alpha-tubulin in HA-treated MDA-MB-231 cells was detected, especially in mitotic cells. Our previous study demonstrated that forced-induced HAS3 overexpression in neuroblastoma formed abnormal cristae. Such results were suggesting that HAS3 plays a critical role in initiating HAS3-induced mitophagy in N2a cells [10]. These studies indicate that HAS3 overexpression in human tumor cells plays a vital role in inhibiting tumor growth. This study explored the anti-migratory effects of HAS3 protein and its enzymatic product, HA, in the ECM surrounding tumor tissues. We also demonstrated the mechanisms of HAS3-induced G2/M cell growth cycle arrest accompanied by autophagic cell death in breast cancer cells. 

## Figures and Tables

**Table 1:** Demographic evaluation of clinical criteria and HAS3 mRNA expression fold changes between tumor vs. normal paired samples.

Characteristic	Total No. of Patients	HAS3 (N > T)			HAS3 (N < T)		
		No.	Mean Fold Change and 95% CI	P	No.	Mean Fold Change and 95% CI	P
				
Age				0.75			0.28
<45 y	73	53	89.49 (16.0 to 163.0)		20	0.26 (0.1 to 0.4)	
≥45y	258	197	109.14 (50.4 to 167.9)		61	0.33 (0.3 to 0.4)	
Nodal status				0.38			0.99
N0	152	119	150.77 (55.1 to 246.4)		33	0.31 (0.2 to 0.4)	
N1	91	69	57.08 (8.7 to 105.4)		22	0.3 (0.2 to 0.4)	
N2	42	33	66.95 (11.7 to 122.2)		9	0.33 (0.1 to 0.6)	
N3	36	25	78.12 (−12.8 to 169.0)		11	0.32 (0.1 to 0.5)	
Stage				0.85			0.03
0	7	5	8.45 (−2.5 to 19.4)		2	0.62 (−0.7 to 1.9)	
I	71	56	104.39 (29.4 to 179.4)		15	0.39 (0.3 to 0.5)	
II	159	119	126.43 (33.4 to 219.4)		40	0.27 (0.2 to 0.3)	
III	91	69	70.25 (24.7 to 115.8)		22	0.29 (0.2 to 0.4)	
IV	2	1	265.1		1	0.89	
ER status				0.76			0.46
Negative	78	60	91.27 (16.5 to 166.1)		18	0.36 (0.2 to 0.5)	
Positive	252	190	109.28 (49.5 to 169.1)		62	0.3 (0.2 to 0.4)	
PR status				0.19			0.75
Negative	137	103	143.78 (34.3 to 253.3)		34	0.33 (0.2 to 0.4)	
Positive	190	145	78.63 (46.1 to 111.2)		45	0.31 (0.2 to 0.4)	
Her-2 status				0.99			0.69
Negative	237	180	105.8 (42.9 to 168.6)		57	0.31 (0.2 to 0.4)	
Positive	77	56	105.54 (24.7 to 186.4)		21	0.34 (0.2 to 0.5)	
Path				0.98			0.22
DCIS	12	8	11.27 (−1.6 to 23.9)		4	0.57 (0.3 to 0.9)	
Infiltration ductal carcinoma	289	218	108.98 (54.7 to 163.3)		71	0.29 (0.2 to 0.4)	
Mucinous carcinoma	8	6	105.15 (−96.2 to 306.5)		2	0.33 (−3.6 to 4.3)	
ILC	13	9	31.75 (0.4 to 63.1)		4	0.35 (−0.2 to 0.9)	
IPC	1	1	2.75		0		
Mix	8	8	182.74 (−228.4 to 593.9)		0		
other	1	1	16.44		0		

ER: estrogen receptor; PR: progesterone receptor; Her-2: human epidermal growth factor receptor 2; DCIS: ductal carcinoma in situ; ILC: invasive lobular carcinoma; IPC: intracystic papillary carcinoma.

**Table 2:** Demographic evaluation of clinical criteria and HAS2 mRNA expression fold changes between tumor vs. normal paired samples.

Characteristic	Total No. of Patients	HAS2 (N > T)			HAS2 (N < T)		
		No.	Mean Fold Change and 95% CI	P	No.	Mean Fold Change and 95% CI	P
				
Age				0.11			0.18
<45 y	48	23	327.2 (−58.0 to 607.7)		25	32.2 (−0.1 to 0.2)	
≥45y	179	77	52.4 (−356.5 to 906.0)		102	432.8 (−0.1 to 0.2)	
Nodal status				0.75			0.53
N0	107	51	197.0 (−242.3 to 537.5)		56	510.9 (−271.5 to 984.7)	
N1	62	26	49.4 (−137.4 to 432.6)		36	154.3 (−170.0 to 883.2)	
N2	24	12	12.9 (−11.5 to 18.7)		12	53.3 (−716 to 175.2)	
N3	25	7	9.3 (−10.7 to 17.9)		18	323.7 (−644.1 to 103.3)	
Stage				0.69			0.31
0	2	0	0		2	46.1 (−3491.5 to 2050.6)	
I	49	20	113.9 (−520.2 to 419.9)		29	766.5 (−2050.6 to 3491.5)	
II	116	55	164.1 (−419.9 to 520.2)		61	264.4 (−2505.5 to 2942)	
III	45	23	10.8 (−653.6 to 447.2)		22	191.0 (−2613.0 to 2902.7)	
IV	0	0	0		0	0	
ER status				0.47			0.76
Negative	47	21	16.0 (−473.9 to 221.6)		26	284.3 (−677.4 to 494.9)	
Positive	179	79	142.2 (−305.5 to 53.3)		100	375.5 (−561.7 to 379.2)	
PR status				0.39			0.32
Negative	86	43	45.9 (−408.1 to 163.5)		43	193.9 (−753.7 to 249.3)	
Positive	139	57	168.3 (−374.4 to 129.7)		82	446.1 (−662.9 to 158.6)	
Her-2 status				0.59			0.26
Negative	195	88	130.1 (−315.9 to 556.7)		107	411.6 (−277.2 to 1009.2)	
Positive	32	12	9.8 (−40.8 to 281.5)		20	45.5 (82.2 to 649.8)	
Path				0.99			0.96
DCIS	5	0	0		5	26.2 (−1704.6 to 909.1)	
Infiltration ductal carcinoma	193	89	125.2 (−689.3 to 817.3)		104	423.9 (−684.9 to −110.5)	
Mucinous carcinoma	9	4	61.2 (−130.7 to 258.6)		5	3.7 (−171.4 to 15.2)	
ILC	12	6	29.7 (−97.7 to 153.4)		6	81.8 (−173.9 to 17.8)	
IPC	1	0	0		1	10.9	
Mix	0	0	0		0	0	
other	6	1	1.86		5	39.9 (−219.4 to 161.4)	

ER: estrogen receptor; PR: progesterone receptor; Her-2: human epidermal growth factor receptor 2; DCIS: ductal carcinoma in situ; ILC: invasive lobular carcinoma; IPC: intracystic papillary carcinoma.

**Table 3:** Demographic evaluation of clinical criteria and HAS1 mRNA expression fold changes between tumor vs. normal paired samples.

Characteristic	Total No. of Patients	HAS1 (N > T)			HAS1 (N < T)		
		No.	Mean Fold Change and 95% CI	P	No.	Mean Fold Change and 95% CI	P
				
Age				0.75			0.29
<45 y	52	36	63.9 (−39.7 to 55.1)		16	19.4 (−179.2 to 50.2)	
≥45y	175	113	56.2 (−47.6 to 63.0)		62	83.9 (−124.7 to −4.3)	
Nodal status				0.18			0.59
N0	112	78	66.9 (−42.5 to 87.0)		34	76.5 (−222.7 to 134.5)	
N1	71	53	44.7 (−160.9 to 41.8)		18	120.6 (−181.8 to 258.9)	
N2	27	17	104.3 (−36.9 to 220.6)		10	37.9 (−324.3 to 158.9)	
N3	26	15	12.4 (−157.0 to 47.9)		11	16.3 (−272.7 to 152.3)	
Stage				0.84			0.11
0	3	2	5.9 (−2.5 to 19.4)		1	1.14	
I	53	38	44.3 (29.4 to 179.4)		15	112.7 (−768.1 to 544.9)	
II	123	88	62.2 (33.4 to 219.4)		35	70.1 (−49.4 to 133.9)	
III	64	40	54.8 (24.7 to 115.8)		24	27.8 (−36.4 to 120.9)	
IV	1	0	0		1	566.2	
ER status				0.68			0.77
Negative	52	36	50.0 (−57.3 to 37.5)		16	85.3 (−99.1 to 133.9)	
Positive	194	133	59.9 (−53.5 to 33.7)		61	67.9 (−83.6 to 118.5)	
PR status				0.65			0.53
Negative	90	65	63.7 (−30.8 to 49.4)		25	49.8 (−132.9 to 68.5)	
Positive	155	103	54.4 (−32.1 to 50.6)		52	81.9 (−114.4 to 50.1)	
Her-2 status				0.66			0.89
Negative	209	143	59.7 (−41.9 to 65.7)		66	69.3 (−138.4 to 120.5)	
Positive	38	26	47.7 (−32.0 to 55.9)		12	78.2 (−130.2 to 112.3)	
Path				0.86			0.85
DCIS	6	3	4.4 (−212.7 to 94.7)		3	3.3 (0.3 to 0.9)	
Infiltration ductal carcinoma	212	142	63.5 (−81.9 to −36.1)		70	73.5 (0.2 to 0.4)	
Mucinous carcinoma	7	7	19.0 (−14.9 to 20.1)		0	0	
ILC	13	8	16.5 (−15.2 to 20.3)		5	71.8 (−0.2 to 0.9)	
IPC	1	1	7.45		0		
Mix	0	0	0		0	0	
other	7	7	66.8 (−466.7 to 347.9)		0	0	

ER: estrogen receptor; PR: progesterone receptor; Her-2: human epidermal growth factor receptor 2; DCIS: ductal carcinoma in situ; ILC: invasive lobular carcinoma; IPC: intracystic papillary carcinoma.

## Data Availability

Data is contained within the article or supplementary material.

## Supplementary Materials

The following are available online. Figure S1: The mRNA expression level in different groups of breast tumor tissues; Figure S2: HAS3 induced autophagic regulatory protein expression in MDA-MB-231 cells; Figure S3: The HAS3 derived HA with specific MW was fractionated from the MEF.

## Sample Availability

Samples of the compounds are available from the authors.

## Abbreviations

ECM	extracellular matrix
HA	hyaluronic acid
HAS1	hyaluronan synthases 1
HAS2	hyaluronan synthases 2
HAS3	hyaluronan synthases 3
ABC	ATP-binding cassette
MW	molecular weight
ALL	acute lymphoblastic leukemia
MLL	mixed lineage leukemia
ER	endoplasmic reticulum
TMZ	temozolomide
RIPK1	receptor-interacting protein kinase 1
ROS	reactive oxygen species
HB-TDFs	human breast tumor-derived fibroblasts
TNBC	triple-negative breast cancer
PDX	patient derived xenograft
DMEM/F12	Dulbecco’s Modified Eagle Medium/Nutrient Mixture F-12
RPMI 1640	Roswell Park Memorial Institute 1640
FBS	fetal bovine serum
α-SMA	alpha-smooth muscle actin
MTT	3-(4,5-dimethylthiazol-2-yl)-2,5-diphenyltetrazolium
siRNA	small interfering RNA
scRNA	scrambled siRNA
BLAST	basic local alignment search tool
IHC	immunohistochemistry
PBS	phosphate-buffered saline
DPX	distyrene, plasticizer, xylene
ELISA	enzyme-linked immunosorbent assay
MEF	mouse embryonic fibroblasts
MEF-Has3-KO	HAS3 knockout mouse embryonic fibroblasts
PCR	polymerase chain reaction
GAPDH	glyceraldehyde 3-phosphate dehydrogenase
HCl	hydrochloric acid
NaCl	sodium chloride
EDTA	ethylenediaminetetraacetic acid
EGTA	ethylene glycol tetraacetic acid
NaF	sodium fluoride
SDS-PAGE	sodium dodecyl sulfate-polyacrylamide gel electrophoresis
BSA	bovine serum albumin
TEM	transmission electron microscopy
ASW	artificial seawater
PI	propidium iodide
FLIM	fluorescence lifetime imaging
EYFP	enhanced yellow fluorescent protein
CRISPR	clustered regularly interspaced short palindromic repeats
Cas9	CRISPR-associated protein 9
sgRNAs	single guide RNAs
tracrRNA	transactivating RNA
RNP	ribonucleoprotein
SCID	severe combined immunodeficient
IVIS	in vivo imaging system
NSG	non-obese diabetic–severe combined immunodeficiency–iL2Rgammanull
PR	progesterone receptor
HER2	human epidermal growth factor receptor 2
