# Supplementary material for: Type-3 Hyaluronan Synthase Attenuates Tumor Cells Invasion in Human Mammary Parenchymal Tissues
Source: Molecules. 2021 Oct 29;26(21):6548. doi: 10.3390/molecules26216548 (PMC8587416; doi:10.3390/molecules26216548)
Supplement: Supplementary file 1 [file molecules-26-06548-s001.zip › molecules-1399990-supplementary.pdf]

Type-3 Hyaluronan Synthase Attenuates Tumor Cells Invasion in Human Mammary Parenchymal Tissues

Wen-Jui Lee [1,2], Shih-Hsin Tu [3,4,5], Tzu-Chun Cheng [6], Juo-Han Lin [7], Ming-Thau Sheu [8], Ching-Chuan Kuo [9],Chun A. Changou [10,11,12], Chih-Hsiung Wu [13], Hui-Wen Chang [14] Hang-Lung Chang [15], Li-Ching Chen [3,4,16,*] and Yuan-Soon Ho [6,14,16,17,18,*]

1 Ph.D. Program for Neural Regenerative Medicine, College of Medical Science and Technology, Taipei Medical University, Taipei 110,Taiwan; sam19871119@hotmail.com
2 Ph.D. Program for Neural Regenerative Medicine, College of Medical Science and Technology, National Health Research Institutes. Miaoli County 350, Taiwan
3 Breast Medical Center, Taipei Medical University Hospital, Taipei 110, Taiwan; drtu@h.tmu.edu.tw
4 Taipei Cancer Center, Taipei Medical University, Taipei 110, Taiwan
5 Department of Surgery, School of Medicine, College of Medicine, Taipei Medical University, Taipei 110, Taiwan
6 Graduate Institute of Medical Sciences, College of Medicine, Taipei Medical University, Taipei 110, Taiwan; d119096007@tmu.edu.tw
7 Program for Cancer Molecular Biology and Drug Discovery, College of Medical Science and Technology, Taipei Medical University and Academia Sinica, Taipei 110, Taiwan; d621100001@tmu.edu.tw
8 Department of Pharmaceutical Sciences, Taipei Medical University, Taipei 110, Taiwan; mingsheu@tmu.edu.tw
9 Institute of Biotechnology and Pharmaceutical Research, National Health Research Institutes, Zhunan 350, Taiwan; cckuo@nhri.org.tw
10 The PhD Program for Translational Medicine, College of Medical Science and Technology, Taipei Medical University, Taipei 110, Taiwan; austinc99@tmu.edu.tw
11 The PhD Program for Cancer Molecular Biology and Drug Discovery, College of Medical Science and Technology, Taipei Medical University and Academia Sinica, Taipei 110, Taiwan
12 The Core Facility Center, Office of Research and Development, Taipei Medical University, Taipei 110, Taiwan
13 Department of General Surgery, En Chu Kong Hospital, New Taipei City 110 Taiwan; chwu@tmu.edu.tw
14 Department of Laboratory Medicine, Taipei Medical University Hospital, Taipei 110, Taiwan; g160090005@tmu.edu.tw
15 Department of General Surgery, En Chu Kong Hospital, New Taipei City 237, Taiwan; changhl0321@gmail.com
16 TMU Research Center of Cancer Translational Medicine, Taipei Medical University, Taipei 110, Taiwan
17 Graduate Institute of Neural Regenerative Medicine, College of Medical Science and Technology, Taipei Medical University, Taipei 110, Taiwan
18 School of Medical Laboratory Science and Biotechnology, College of Medical Science and Technology, Taipei Medical University, Taipei 110, Taiwan
* Correspondence: lcchen@tmu.edu.tw (L.-C.C.); hoyuansn@tmu.edu.tw (Y.-S.H.)

Supplementary Figures

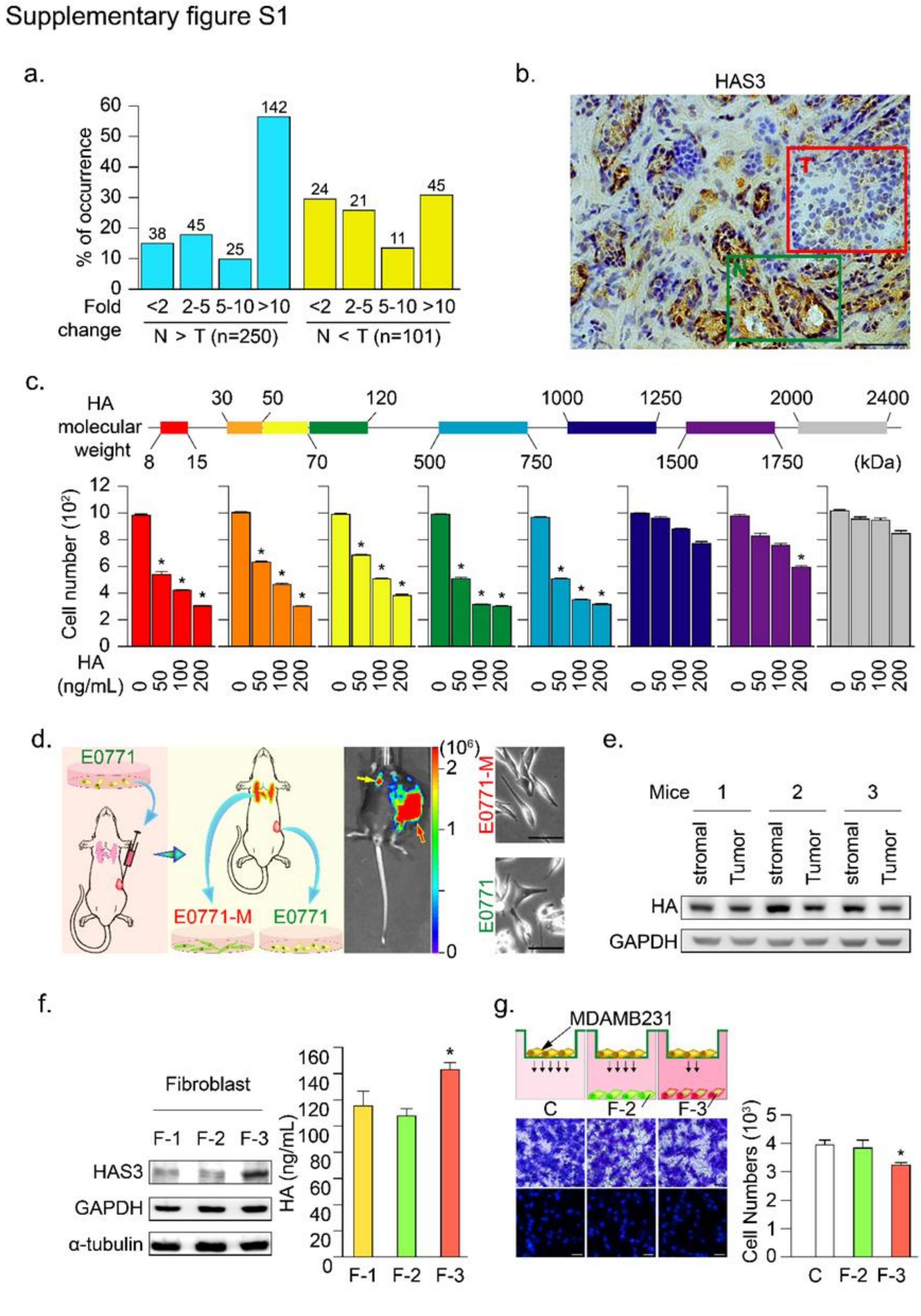

Figure S1. The mRNA expression level in different groups of breast tumor tissues. (**a**) The paired normal, and tumor tissue samples with different levels of HAS3 mRNA in Figure 1a (left panel) were subdivided into four groups depending on the magnitude of the difference in expression (<2-, 2–5-, 5–10-, and >10-fold). (**b**) Immunolocalization of the HAS3 protein in human breast tumor tissues. Table 3. N, normal; T, tumor; IHC, immunohistochemistry. Normal breast epithelial cells are

indicated by a green rectangle, whereas a red rectangle indicates cancer cells. Scale bar = 100 μm. (**c**) HA inhibits the migratory activity of human breast cancer lines (MDA-MB-231). A Transwell assay was conducted for the migration assay. The HA (50–200 ng/mL) was applied to the lower chamber, and the migratory activity of breast cancer (MDA-MB-231) cells cultured in the upper chamber was evaluated. The data shown indicate the number of migrated human breast cancer cells. Error bars indicate 95% confidence intervals. Data were analyzed with a paired t-test, and the presented P-values are two-sided. *$P < 0.05$ compared with the control group. (**d**) The E0771 cells were transfected with a FLUC-pcDNA3.1 plasmid and transplanted subcutaneously into the backs of C57B6/J mice. Tumor migration was monitored through the IVIS200 system weekly until six weeks after cell transplantation. The morphology of E0771 and E0771-M cells was presented. Scale bar = 100 μm. (**e**) The protein lysates were harvested from patient-derived xenografted (PDX) tumor and the stroma tissues from NSG mice, and the protein level of HA was determined by western blotting analysis. The GAPDH protein level was detected as a control to ensure equal protein loading. (**f**) Left, the HAS3 protein level in these cells was detected by western blotting in three HB-TDFs (denoted as F-1, F-2, and F-3). Middle, HA levels in the conditioned medium of cultured HB-TDFS cells were determined. The GAPDH protein level was detected as a control to ensure equal protein loading. The data are presented as the mean ± SD; *$P < 0.05$ compared with the control group. (**g**) HB-TDFS-F-2 and HB-TDFS-F-3 cells were selected as representative groups of cells with different HAS3 protein levels (low vs. high, respectively). A Transwell assay using MDA-MB-231 cells (upper chamber) cocultured with HB-TDFs (lower chamber) was carried out. Quantitative data are shown in Figure 1h, lower panel. Magnification, 200×; scale bar, 50 μm. The data are presented as the mean ± SD; $P < 0.05$ compared with the control group.

Supplementary figure S2

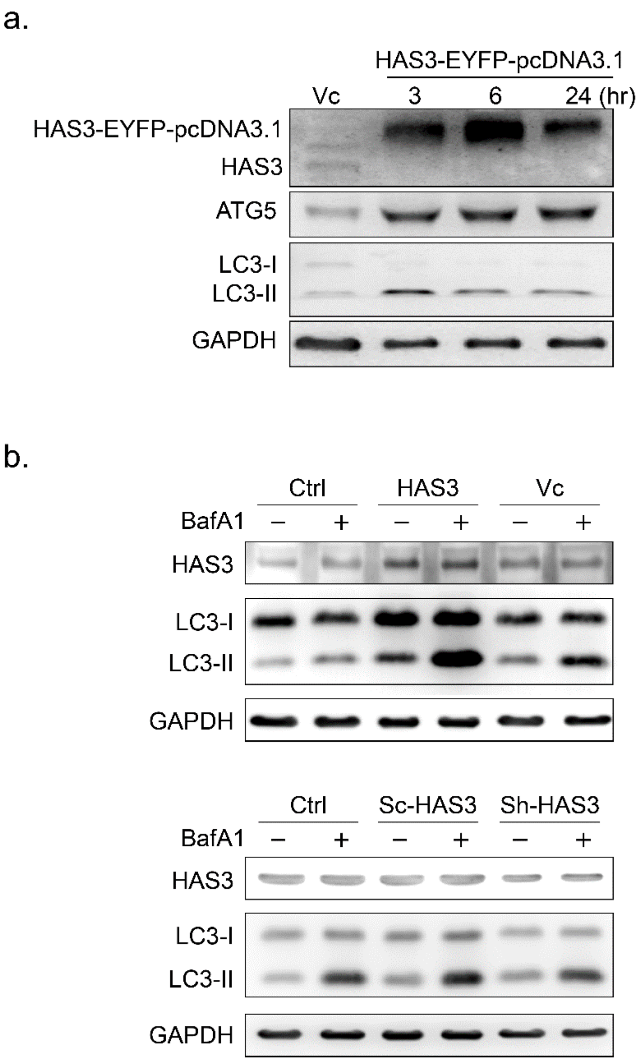

Figure S2. HAS3 induced autophagic regulatory protein expression in MDA-MB-231 cells. (**a**) HAS3-EYFP-pcDNA3.1-transfected MDA-MB-231 cells were harvested and assessed for autophagy-associated protein expression (ATG5 and LC3 conversion) by western blot analysis. Cells transfected with the EYFP plasmid were used as a control. GAPDH was detected as a protein loading control. (**b**) Overexpression of HAS3-induced autophagic regulatory protein expression was attenuated by an autophagic specific inhibitor (BafA1, 50 nM, 24 h) in MDA-MB-231 cells. Protein lysates were harvested and assessed for autophagy-associated protein expression (LC3 conversion) by western blot analysis. GAPDH was detected as a protein loading control.

Supplementary figure S3

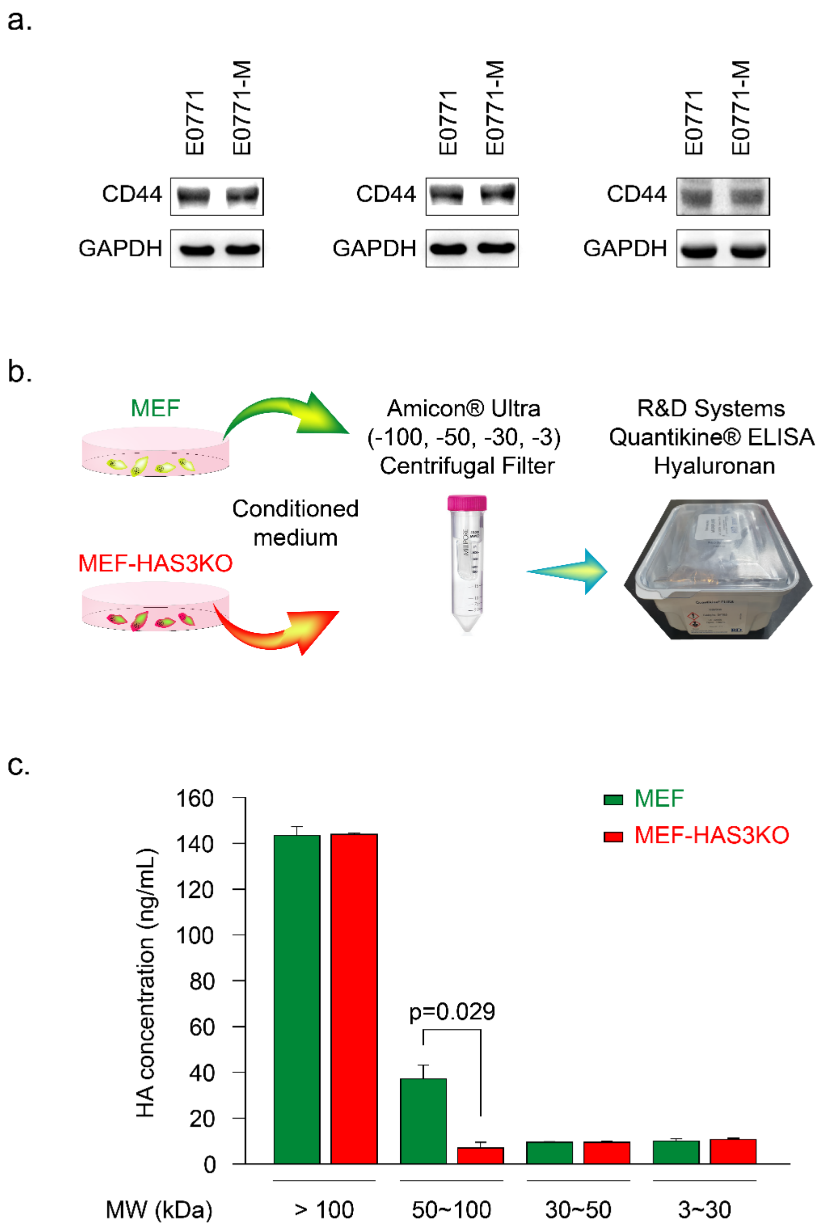

Figure S3. The HAS3 derived HA with specific MW was fractionated from the MEF. (**a**) The protein expression of CD44 in mouse breast cell lines (E0771 and E0771-M) was detected by immunoblotting. The GAPDH protein level was detected as a control to ensure equal protein loading. (**b**) Workflow of the methodology used to concentrate MEF and MEF-Has3-KO conditioned medium by Amicon Ultra centrifugal filtration. HA levels in the conditioned medium of cultured MEFs and MEF-Has3-KO cells were determined by using an HA ELISA kit. (**c**) An ELISA reader detected quantitative data of HA concentration. The data are presented as the mean ± SD; $P < 0.05$ compared with the MEF group.
